# Soil Quality Indexing Strategies for Evaluating Sugarcane Expansion in Brazil

**DOI:** 10.1371/journal.pone.0150860

**Published:** 2016-03-03

**Authors:** Maurício R. Cherubin, Douglas L. Karlen, Carlos E. P. Cerri, André L. C. Franco, Cássio A. Tormena, Christian A. Davies, Carlos C. Cerri

**Affiliations:** 1 University of São Paulo, “Luiz de Queiroz” College of Agriculture, Department of Soil Science, Piracicaba, São Paulo, Brazil; 2 United States Department of Agriculture—Agricultural Research Service, National Laboratory for Agriculture and the Environment, Ames, Iowa, United States of America; 3 Colorado State University, Department of Biology, Fort Collins, Colorado, United States of America; 4 State University of Maringá, Department of Agronomy, Maringá, Paraná, Brazil; 5 Shell Technology Center Houston, Houston, Texas, United States of America; 6 University of São Paulo, Center for Nuclear Energy in Agriculture, Piracicaba, São Paulo, Brazil; Chinese Academy of Sciences, CHINA

## Abstract

Increasing demand for biofuel has intensified land-use change (LUC) for sugarcane (*Saccharum officinarum*) expansion in Brazil. Assessments of soil quality (SQ) response to this LUC are essential for quantifying and monitoring sustainability of sugarcane production over time. Since there is not a universal methodology for assessing SQ, we conducted a field-study at three sites within the largest sugarcane-producing region of Brazil to develop a SQ index (SQI). The most common LUC scenario (i.e., native vegetation to pasture to sugarcane) was evaluated using six SQI strategies with varying complexities. Thirty eight soil indicators were included in the total dataset. Two minimum datasets were selected: one using principal component analysis (7 indicators) and the other based on expert opinion (5 indicators). Non-linear scoring curves were used to interpret the indicator values. Weighted and non-weighted additive methods were used to combine individual indicator scores into an overall SQI. Long-term conversion from native vegetation to extensive pasture significantly decreased overall SQ. In contrast, conversion from pasture to sugarcane had no significant impact on overall SQ at the regional scale, but site-specific responses were found. In general, sugarcane production improved chemical attributes (i.e., higher macronutrient levels and lower soil acidity); however it has negative effects on physical and biological attributes (i.e., higher soil compaction and structural degradation as well as lower soil organic carbon (SOC), abundance and diversity of macrofauna and microbial activity). Overall, we found that simple, user-friendly strategies were as effective as more complex ones for identifying SQ changes. Therefore, as a protocol for SQ assessments in Brazilian sugarcane areas, we recommend using a small number of indicators (e.g., pH, P, K, Visual Evaluation of Soil Structure -VESS scores and SOC concentration) and proportional weighting to reflect chemical, physical and biological processes within the soil. Our SQ evaluations also suggest that current approaches for expanding Brazilian sugarcane production by converting degraded pasture land to cropland can be a sustainable strategy for meeting increasing biofuel demand. However, management practices that alleviate negative impacts on soil physical and biological indicators must be prioritized within sugarcane producing areas to prevent unintentional SQ degradation over time.

## Introduction

Increasing global demand for biofuel has accelerated land-use change (LUC) to support bioenergy crops in many countries. In Brazil, the area devoted to sugarcane production increased from 5.8 to 9.0 Mha during the last decade [1]. Even though Brazil is already the world’s largest sugarcane producer, current predictions indicate that an additional 6.4 Mha of sugarcane will be needed to meet the domestic demand for ethanol by 2021 [2]. Sugarcane expansion has primarily occurred on lands previously occupied by extensive pastures [2, 3], most of which are degraded or in the process of being degraded [4, 5]. To obtain long-term energy security, bioenergy systems will need to be agronomically and environmentally sustainable. Intensification of land use through mechanization and agrochemical inputs has direct implications on soil physical, chemical and biological properties and consequently on the quality/health of soils. To prevent unintended consequences, monitoring of soil property changes due to LUC is essential [6, 7]. However, this research topic is still new in Brazil, and we are not aware of any protocol for evaluating soil quality (SQ) changes induced by sugarcane expansion in this region.

Soil quality was defined as the capacity of a specific kind of soil to function, within natural or managed ecosystem boundaries, to sustain plant and animal productivity, maintain or enhance water and air quality, and support human health and habitation [8]. It is a complex functional concept and cannot be measured directly in the field or laboratory; but can be indirectly inferred by soil indicators [8, 9]. Indicators of SQ are those measurable soil properties and processes that have greatest sensitivity to changes in soil function and its ecosystem services [7, 10]. A wide range of soil chemical, physical and biological properties could be measured [7, 11–14], but due to cost it’s not feasible to consider them all, and therefore it is necessary to select a minimum dataset (MDS). Several strategies have been used to define an appropriate MDS including principal component analysis (PCA) [9, 15–20], fuzzy sets [21–22], expert opinion [10, 12] and farmer/local knowledge [23, 24]. According Doran and Parkin [25] suitable SQ indicators should encompass ecosystem processes, integrate soil properties, be accessible to many users, sensitive to management and climate, and, whenever possible, be components of existing databases. An example for reducing the number of potential SQ indicators was provided by Andrews et al. [10] through their development of the Soil Management Assessment Framework (SMAF). Starting with an extensive list of 80 or more integrative measurements related to ecosystem processes and functions that reflect SQ, they developed scoring curves only for a small number (i.e., 10) of carefully selected indicators that could reliably detect SQ changes induced by agricultural management practices. In more recent studies, others have shown that small datasets can effectively characterize SQ within different ecosystems. Lima et al. [26] compared SQ assessment using a total dataset (TDS) of 29 indicators, a MDS of eight indicators based on PCA, and an indigenous set of four indicators based on farmer knowledge to evaluate rice (*Oryza sativa* L.) production systems in southern Brazil. They concluded that the TDS provided the best assessment of SQ, but the smaller datasets showed the same SQ trends and thus provided meaningful information for land managers. Askari and Holden [18, 19] reduced the number of indicators using PCA from 21 to 3 and from 22 to 7, respectively, and verified that the MDS indicators were suitable to efficiently quantify SQ in grassland and arable fields in Ireland.

After defining a MDS, linear and non-linear techniques, each with their advantages and disadvantages, have been applied to interpret SQ indicators [12, 18, 19, 27]. While linear methods are simple, user-friendly and require little knowledge of the indicator thresholds, non-linear methods can more often assign meaningful scores that better represent the soil functions being represented by the indicators [12].

Once individual indicators have been scored, it is often convenient, but not essential, to integrate them into an overall SQ index (SQI) that can be used to support decision making and selection of sustainable management practices [28]. Currently, there is no comprehensive, universal SQI that can be used across multiple natural and anthropogenic ecosystems. Many indexing strategies have been developed and tested for specific purposes under particular environmental conditions around the world (e.g., in the U.S.A. [9, 10, 12, 28–30], Brazil [26], Argentina [31], Italy [16], Spain [20], Ireland [18, 19], South Africa [32], India [27] and China [17, 21, 33]). The most user-friendly method to calculate a SQI is to simply add all indicator scores and then divide by the number of indicators [9, 10, 12, 29]. The major concern regarding this method is that when the number of indicators is unbalanced among chemical, physical and biological sectors, the overall SQI misrepresents the sector(s) having fewer indicators. On the other hand, several studies have used methods that assign weights for each indicator. Different criteria that have been used include soil function frameworks [9, 11, 26], principal component loading [9, 12, 20, 27, 33], partial least squares regression coefficients [28] and correlation with crop yield [30]. Simple and weighted additive SQ indexing strategies provide site-specific responses [9, 12, 18, 19], influenced by existing dataset, soil type, and effects of land use and management practices.

Developing more user-friendly and cost-effective strategies for assessing SQ changes induced by agricultural management practices, especially those associated to bioenergy feedstock production therefore remains a challenge for the scientific community [9, 10, 12, 28]. Our goal was to develop a sensitive and reliable protocol for evaluating SQ impact associated with LUC occurring to increase Brazilian sugarcane production. To do so, we conducted a field-study at three sites where the primary LUC sequence (i.e., native vegetation to pasture to sugarcane) is occurring within of the largest sugarcane-producing region of Brazil. Six SQ indexing strategies with varying complexity were developed and tested. Our hypotheses were that: (i) the LUC sequence would result in SQ degradation; (ii) the SQI approach would be suitable to detect SQ changes due to LUC; and (iii) the simple, more user-friendly strategies would be able to detect SQ changes as effectively as more complex strategies.

## Material and Methods

### Field sites and experimental design

**Fig 1:**
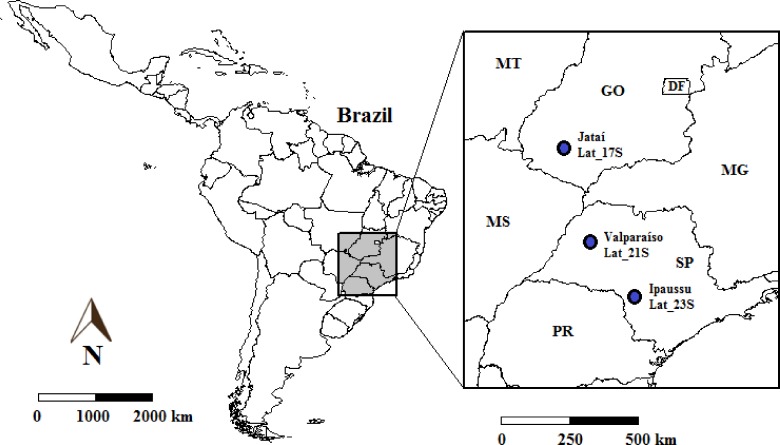
Geographic location of study sites in central-southern Brazil.

Land-use change effects on SQ were evaluated at three sites along a 1000-km transect within central-southern Brazil (Fig 1), which is the largest sugarcane-producing region in the world. Soil samples were collected from each LUC phase at: (i) Lat_17S located in southern Goiás state, the largest hotspot of sugarcane expansion in Brazil; (ii) Lat_21S located in western São Paulo state, a transition area between traditional and new sugarcane production cores, and (iii) Lat_23S: located in south-central São Paulo state, which represents the traditional sugarcane production core in Brazil.

Climate patterns at all three sites are similar with rainfall concentrated in the spring and summer (October to April) followed by a dry season in autumn and winter (May to September). The soils are typical for the Brazilian tropical region, well-drained and highly weathered, classified as Oxisols (Lat_17S and Lat_23S) or Alfisols and Ultisols (Lat_21S). A chronosequence was sampled at each site representing the three land uses (native vegetation, pasture and sugarcane) associated with the most common LUC sequence in central-southern Brazil. The synchronic approach (chronosequence) was chosen to represent potential long-term changes occurring in the region due to this LUC. The land use history involves conversion of the native vegetation (Cerrado and Atlantic Forest) to extensive pasturelands in the beginning of 1980’s. These areas are typical Brazilian pasturelands, which are cropped with tropical grasses (*Brachiaria* and *Cynodon* genus) and characterized by absence of management, extensive and continuous grazing and low grass productivity and stocking rates (i.e., 1 animal unit ha^-1^). Sugarcane was subsequently established on a portion of the pasture during the early 1990s at Lat_23S, and more recently in 2009 and 2010 at Lat_17S and Lat_21S. Conversion to sugarcane required intensive, heavy tillage operations (plowing and disking) and the application of lime. Subsequent annual sugarcane management requires fertilization using mineral and/or organic fertilizers, and pesticide applications to control weed, pests and diseases. Traditionally, sugarcane fields were burned before harvest, but at the Lat_23S site, the crop has been mechanically harvested without any burning since 2003 and at the Lat_17S and Lat_21S sites, the crop was never burned. Additional location, climate, soil, land use and management information for each site is available in Cherubin et al. [34].

### Soil sampling and analysis

All soil samples were collected using a consistent experimental design that had four points spaced 50 m apart imposed within each land use. This provided 12 sampling points for each location or 36 sampling points for the three locations. A small trench (30 x 30 x 30 cm) was opened at each sampling point to collect both undisturbed and semi-undisturbed samples from the 0 to 10-, 10 to 20- and 20 to 30-cm layers. This provided 108 samples for physical analyses, and 108 for soil aggregation and macrofauna analyses. An additional 108 disturbed samples were collected for chemical and biological analyses by compositing 12 subsamples taken from each soil layer with a Dutch auger.

Available phosphorus (P), potassium (K), calcium (Ca), magnesium (Mg), sulfur (S—sulphate), boron (B), cooper (Cu), manganese (Mn), iron (Fe), zinc (Zn), active acidity (pH_CaCl2_ 0.01mol L^-1^), potential acidity (H+Al), base saturation (BS) and potential cation exchange capacity (CEC_pH7_) were determined using analytical methods described by Raij et al. [35]. Soil resistance to penetration (SRP) and field-saturated hydraulic conductivity (K_fs_) were measured at five and three locations, respectively, within ~5 m of each trench using a digital penetrometer (PenetroLOG^®^) and the ‘simplified falling-head’ method proposed by Bagarello et al. [36]. Soil structural quality of the 20 x 10 x 25 cm monoliths from each trench was assessed using the Visual Evaluation of Soil Structure (VESS) method [37, 38]. Particle-size was determined using the hydrometer method. Bulk density (BD) was determined using the core method with 100 cm^3^ cylinders. Soil degree of compactness (SDC) was calculated as SDC = (BD/BD_max_) x 100, where BD_max_ is maximum bulk density, estimated using the pedotransfer function described by Marcolin and Klein [39]. Total porosity (TP) was calculated as TP = 1 - (BD/PD), where, PD is particle density, determined using a gas pycnometer. Soil water content at -6 kPa and -10 kPa water potential was determined using tension tables as described by Ball and Hunter [40]. Soil macroporosity (MaP) was computed as the difference between soil water content at saturation and at -6 kPa. Soil microporosity (MiP) was estimated as the soil water content at -6 kPa. Water-filled pore space (WFPS) was calculated by dividing volumetric moisture at -6 kPa by total porosity as indicated in Wienhold et al. [41]. We also calculated two indexes suggested by Reynolds et al. [42]: i) soil water storage capacity (SWSC) defined as the ratio between water content at field capacity (FC, -10 kPa soil water potential) and TP (SWSC = FC/TP); and ii) soil aeration capacity (SAC) calculated as the ratio between drained pores at soil water potential of -10 kPa (ACt) and TP (SAC = ACt/TP). A structural stability index (SSI) was calculated as suggested by Reynolds et al. [43]: SSI = ((SOC x 1.724) / (silt + clay))*100. Wet macroaggregate stability (AGS) was determined using a vertical oscillator (Yoder, model MA-148) with three sieves (2000, 250, and 53 μm) moving at a speed of 30 oscillations per min for 10 min. Percentage of macroaggregates was calculated by summing aggregate mass for >2000 and 250 μm classes, dividing by the total soil mass, and multiplying by 100. Mean weight diameter (MWD) was calculated as the sum of the proportion of aggregates in each size fraction, with each proportion weighted by the mean diameter of aggregates in that size fraction. Soil organic carbon (SOC) and total nitrogen (TN) were determined by dry combustion on a LECO^®^ CN-2000 elemental analyzer (furnace at 1350°C in pure oxygen). Carbon and nitrogen within microbial biomass (MBC and MBN) were measured by fumigation/extraction as proposed by Vance et al. [44]. Enzymatic activities of β-Glucosidase (BG) and acid phosphatase (AcP) were measured as described by Tabatabai [45]. Immediately after the sampling, soil macrofauna were carefully hand-sorted from each 25 x 25 x 30 cm soil block, according to the standard Tropical Soil Biology and Fertility Institute (TSBF) soil monolith method [46]. Invertebrates were classified into the taxonomic groups: Aranae, Blattodea, Chilopoda, Coleoptera, Dermaptera, Diplopoda, Diptera, Formicidae, others Hymenoptera, Gastropoda, Hemiptera, Isopoda, Isoptera, Oligochaeta, and Scorpiones. Macrofauna density was determined as the number of individuals per surface unit (m^2^). Ecological indexes were calculated for assessing richness (Margalef’s index) and diversity (Shannon’s index), according to the methods described by Magurran [47].

### Developing the soil quality indexes

**Fig 2:**
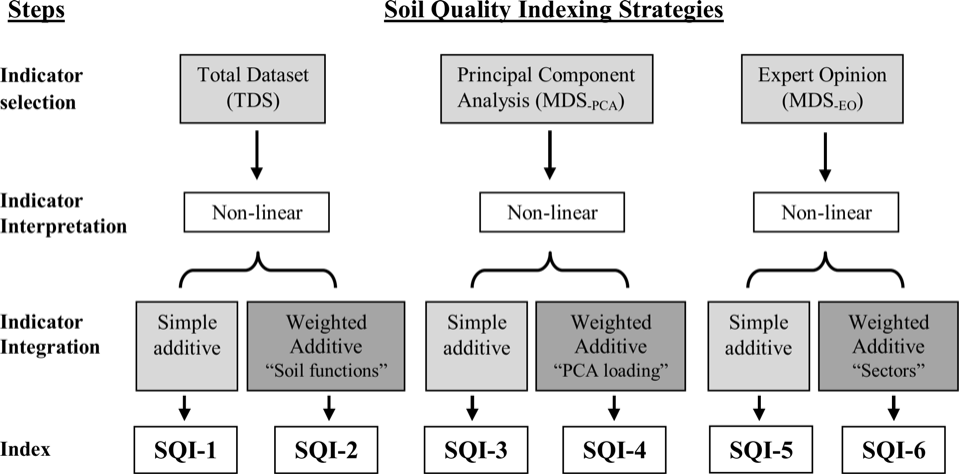
Process diagram for the development of soil quality indexes tested in this study.

Six SQI values were developed using different approaches (Fig 2), although each involved three common steps: selection of SQ indicators as an MDS, transformation of indicator values into unitless 0 to 1 scores using scoring curves, and integration into an overall index [10–13]. The SQIs were compared to identify the most appropriate strategy for assessing SQ changes induced by LUC associated with sugarcane expansion in Brazil. Soil data from the 0 to 10-, 10 to 20- and 20 to 30-cm layers were averaged to create a 0 to 30-cm layer that was then used to calculate an overall SQI that better represented the whole soil profile.

#### Step 1- Indicator selection

**Fig 3:**
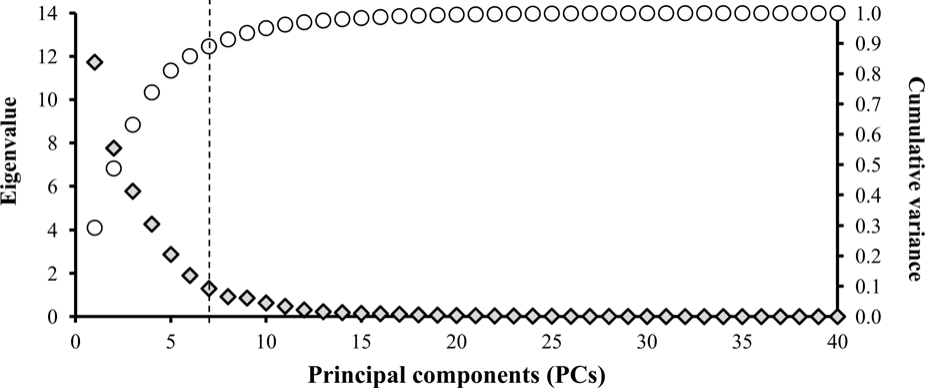
Scree plot of principal component analysis.

Three indicator selection approaches were evaluated: (i) the Total Dataset (TDS) which included 38 indicators representing 14 chemical, 14 physical and 10 biological properties and processes; (ii) a MDS_-PCA_ created using PCA on the TDS to reduce data redundancy and identify the most efficient indicators, without depending upon subjective, expert opinion or literature values, and (iii) a five indicator MDS_-EO_ chosen based on expert opinion and literature review. For the MDS_-PCA_, only seven components with eigenvalues >1 (Kaiser’s criteria) were retained and subjected to varimax rotation to enhance the interpretability of the components (Fig 3). Furthermore, for each component, only the indicators with loading values within 10% of the highest value were retained [12, 17–19, 33]. When more than one indicator was retained, correlation values among them were analyzed (S1 Table). If the indicators were significantly correlated (*p*<0.01), only the one with the highest loading factor was retained in the MDS to avoid redundancy [9, 12, 17–19]. The MDS_-EO_ was selected taking into account the indicator’s ability to detect soil function changes as well as the ease, practicality and cost-effectiveness for sampling, analysis and interpretation.

#### Step 2- Indicator interpretation

**Fig 4:**
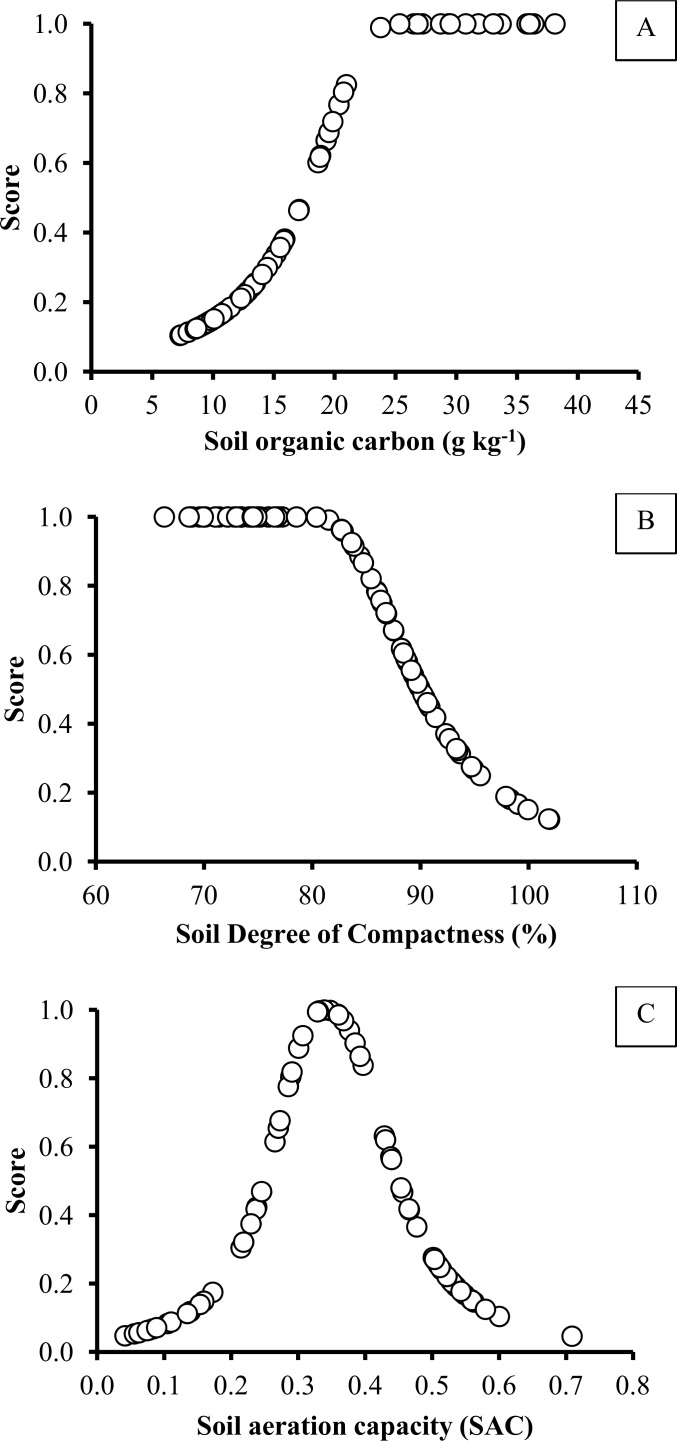
Examples of the scoring curve shapes used for scoring each soil quality indicator. A) more-is-better function; B) less-is-better function; C) mid-point optimum function.

All measured indicator values were transformed using non-linear scoring functions. Based on agronomic and environmental soil functions, each indicator was scored using one of the following curves: “more is better” (upper asymptote sigmoid curve), “less in better” (lower asymptote sigmoid curve), and “mid-point optimum” (Gaussian curve), as exemplified in Fig 4. The non-linear Eqs 1 and 2 were used for “more is better” and “less is better” scoring curve shapes, respectively. For “mid-point optimum” curve the Eqs 1 and 2 were jointly used in the increasing and decreasing parts of the curve, respectively.

$$\text{Score}=\frac{a}{\left[1+{\left(\frac{\text{B}-\text{UB}}{x-\text{UB}}\right)}^{\text{S}}\right]}$$ (1)

$$\text{Score}=\frac{a}{\left[1+{\left(\frac{\text{B}-\text{LB}}{x-\text{LB}}\right)}^{\text{S}}\right]}$$ (2)

where, Score is the unitless value of the soil indicator which ranging from 0 to 1, *a* is the maximum score which was equal to 1 in this study, B is the baseline value of the soil indicator where the score equals 0.5, LB is the lower threshold, UB is the upper threshold, *x* is the measured soil indicator value, and S is the slope of equation set to -2.5.

Threshold and baseline values for each soil indicator were based on literature references and expert’s opinion, as presented in the Table 1. Indicator scoring calculations were performed using a Microsoft Excel^®^ spreadsheet.

**Table 1:** Indicator thresholds and scoring curves.

Indicator^§^	Unit	Lower Threshold	Lower Baseline	Upper Threshold	Upper Threshold	Optimum point	Scoring curve	Reference
**Chemical**								
P	mg dm^-3^	2.0	8.0	16.0			More is better	[66]
S	mg dm^-3^	2.5	5.0	10.0			More is better	[66]
K	mmol_c_ dm^-3^	0.4	0.8	1.6			More is better	[66]
Ca	mmol_c_ dm^-3^	2.0	4.0	8.0			More is better	[66]
Mg	mmol_c_ dm^-3^	2.0	4.0	7.0			More is better	[66]
B	mg dm^-3^	0.1	0.3	0.6			More is better	[66]
Cu	mg dm^-3^	0.1	0.4	0.8			More is better	[66]
Fe	mg dm^-3^	2.0	5.0	12.0			More is better	[66]
Mn	mg dm^-3^	0.6	2.5	5.0			More is better	[66]
Zn	mg dm^-3^	0.3	0.6	1.2			More is better	[66]
CEC_pH7_	mmol_c_ dm^-3^	50.0	75.0	150.0			More is better	[67]
H+Al	mmol_c_ dm^-3^	40.0	80.0	100.0			Less is better	[26]
pH _CaCl2_	unitless	4.0	4.5	8.0	7.5	5.5	Optimum	[66]
BS	%	20.0	40.0	80.0			More is better	[66]
**Physical**								
BD*	Mg m^-3^	1.1/1.3/1.5	1.25/1.45/1.65	1.4/1.6/1.8			Less is better	[68]
SDC	%	80.0	90.0	100.0			Less is better	[69]
SRP	MPa	2.0	3.0	5.0			Less is better	[70]
MaP	m^3^ m^-3^	0.05	0.075	0.15			More is better	[42]
MiP	m^3^ m^-3^	0.15	0.20	0.35			More is better	EO‡
TP	m^3^ m^-3^	0.20	0.35	0.50			More is better	EO
WFPS	unitless	0.15	0.30	0.90	0.80	0.60	Optimum	[41]
SWSC	unitless	0.30	0.45	0.90	0.80	0.66	Optimum	[42]
SAC	unitless	0.15	0.25	0.55	0.45	0.34	Optimum	[42]
K_fs_	cm h^-1^	2.0	7.5	15.0			More is better	[71]
AGS	%	0.2	0.4	0.8			More is better	EO
MWD	mm	0.5	1.5	3.0			More is better	[72]
VESS	score	1.5	3.5	5.0			Less is better	[37, 38]
SSI	%	5.0	7.0	9.0			More is better	[43]
**Biological**								
SOC	g kg^-1^	10.0	17.5	25.0			More is better	[73]
TN	g kg^-1^	1.0	1.75	2.5			More is better	EO
MBC	mg kg^-1^	200	275	350			More is better	[73]
MBN	mg kg^-1^	20	27.5	35			More is better	EO
BG	mg kg^-1^ h^-1^	60	90	120			More is better	[73]
AcP	mg kg^-1^ h^-1^	75	100	150			More is better	EO
Eworm	indiv m^-2^	25	100	200			More is better	[74]
MDens	indiv m^-2^	50	200	400			More is better	EO
MRich	unitless	0.0	0.5	1.0			More is better	EO
MDiver	unitless	0.4	0.8	1.6			More is better	EO

^§^P: phosphorus, S: sulfur, K: potassium, Ca: calcium, Mg: magnesium, B: boron, Cu: cooper, Fe: iron, Mn: manganese, Zn: zinc, CEC_pH7_: potential cation exchange capacity, H+Al: potential acidity, pH: potential of hydrogen in solution of CaCl_2_ 0.01 mol L^-1^ (1:2.5), BS: base saturation, BD: bulk density, SDC: soil degree of compactness, SRP: soil resistance to penetration, MaP: macroporosity, MiP: microporosity, TP: total porosity, WFPS: water-filled pore space, SWSC: soil water storage capacity, SAC: soil aeration capacity, K_fs_: field-saturated hydraulic conductivity; AGS: macroaggregation (>250μm) stability, MWD: mean weight diameter, VESS: visual evaluation of soil structure, SSI: structural stability index, SOC: soil organic carbon, TN: total nitrogen, MBC: microbial biomass carbon, MBN: microbial biomass nitrogen, BG:β Glucosidase activity, AcP: acid phosphatase activity, Eworm: number of earthworm, MDens: macrofauna density, MRich: macrofauna richness and MDiver: macrofauna diversity.

*Threshold values are variable according to soil texture, in order clay, clay sandy and sandy soils, respectively.

‡EO: expert opinion.

#### Step 3- Indicator integration into an index

The indicator scores were integrated into indexes through two approaches, simple additive (Eq 3) used to calculate SQI-1, SQI-3 and SQI-5 (Fig 2); and weighted additive (Eq 4) used to calculate SQI-2, SQI-4 and SQI-6 (Fig 2).

$${\text{SQI}}_{\text{SA}}\;\overset{n}{\underset{i=1}{\sum }}\frac{Si}{n}$$ (3)

$${\text{SQI}}_{\text{WA}}\;\overset{n}{\underset{i=1}{\sum }}WiSi$$ (4)

where, *Si* is the indicator score, *n* the number of indicators integrated in the index and *Wi* the weighted value of the indicators. For the TDS, the indicators were weighted according to a framework developed based on five soil functions (Table 2), as suggest by Karlen and Stott [11] and later used by Lima et al. [26]. Step by step procedure used for calculate the SQI-2 is shown in the S2 Table. For the MDS_-PCA_, the indicators were weighted according with proportional variation explained by each principal component (i.e., % variance explained by each component divided by total cumulative variance of all components selected for the MDS). For the MDS_-EO_ the indicators were weighted by chemical, physical and biological sectors, in which each one, regardless of number of indicators, had the same weight (33%) in the final index.

**Table 2:** Soil functions framework and indicators^§^ used to develop the SQI-2.

		Indicators					
Soil Functions	Weight	Level 1	Weight	Level 2	Weight	Level 3	Weight
F(i)—Storage, availability and cycling of nutrients	0.20	Nutriente availability	0.40	Macronutrients	0.80	TN	0.20
						P	0.20
						K	0.15
						Ca	0.15
						Mg	0.15
						S	0.15
				Micronutrients	0.20	B	0.20
						Cu	0.20
						Mn	0.20
						Fe	0.20
						Zn	0.20
		Acidity/Al toxicity	0.40	pH	0.25		
				H+Al	0.25		
				BS	0.50		
		Nutrient storage and cycling	0.15	CEC_pH7_	0.40		
				SOM	0.60	SOC	0.50
						MBC	0.25
						MBN	0.25
		Nutrient cycling	0.05	Enzymatic activity	1.00	AcP	0.50
						BG	0.50
F(ii)—Infiltration, storage and availability of water and soil aeration	0.20	Water infiltration	0.25	K_fs_	0.70		
				Correlated indicators	0.30	SOC	0.20
						BD	0.50
						Eworm	0.30
		Water storage and availability	0.25	SWSC	0.50		
				WFPS	0.30		
				MiP	0.10		
				Correlated indicator	0.10	TP	1.00
		Soil aeration	0.50	SAC	0.45		
				MaP	0.45		
				Correlated indicator	0.10	TP	1.00
F(iii)—Sustain biological activity	0.20	SOC	0.10				
		Microbial biomass	0.30	MBC	0.50		
				MBN	0.50		
		Edaphic macrofauna	0.40	Eworm	0.10		
				Mdens	0.20		
				Mrich	0.30		
				Mdiver	0.40		
		Correlated indicators	0.20	SWSC	0.50		
				SAC	0.50		
F(iv)—Sustain plant growth	0.20	VESS	0.20				
		SRP	0.20				
		Soil compaction	0.50	BD	0.50		
				SDC	0.50		
		Correlated indicators	0.10	SOC	0.20		
				AGS	0.40		
				TP	0.40		
F(v)—Ability to resist degradation	0.20	Structural stability	0.60	SSI	0.50		
				AGS	0.25		
				MWD	0.25		
		Water infiltration	0.40	K_fs_	1.00		

^§^Abbreviations are same as Table 1.

#### Sensitivity of SQ indexing strategies

The sensitivity of the SQ indexing strategies for detecting LUC impacts on SQ was calculated using Eq 5, described by Masto et al. [27].

$$\text{Sensitivity}\;\left(\text{S}\right){=\text{SQI}}_{\left(\text{max}\right)}{/\text{SQI}}_{\left(\text{min}\right)}$$ (5)

where, SQI_(max)_ and SQI_(min)_ are the maximum and minimum SQI observed within each SQ indexing strategies.

### Statistical analysis

Data were tested for normality using Shapiro-Wilk’s tests (*p*>0.05). The results indicated that no transformation was required. Principal component analysis (PCA) was performed using PROC FACTOR procedure to select a MDS based on a statistical approach. An analysis of variance (ANOVA) was computed using PROC GLM procedure to test LUC effects on soil indicators and SQI scores. If the ANOVA F statistic was significant (*p*<0.05), the means were compared using Tukey’s test (*p*<0.05). Linear correlations among SQI strategies were verified by Pearson’s correlation analysis using PROC CORR procedure. All statistical procedures were completed using the software Statistical Analysis System–SAS v.9.3 (SAS Inc, Cary, USA).

### Ethics statement

All locations are farmer-owned, so before collecting samples we received authorization for fieldwork from each landowner, and verified that no endangered or protected species were located at the sites. Therefore, no formal permissions were needed from regulatory agencies.

## Results and Discussion

### Soil quality indicators

Land-use change effects on the 38 soil quality indicators at each site are presented in Table 3. As typically reported for tropical soils, native vegetation sites were characterized by high acidity, low levels of soil organic matter (SOM) and plant-available macronutrients, suitable soil physical conditions, and high activity as well as diversity of edaphic fauna. Long-term conversion from native vegetation to extensive pasture significantly increased soil acidification (i.e., decreased pH and increased H+Al concentrations), depleted SOM (SOC and TN), available macronutrients, B and CTC_pH7_ and, increased micronutrient (Cu, Fe, Mn and Zn) availability. Poor long-term management, which typically includes continuous grazing without liming and/or applying fertilizer over time [4, 5, 34], is a major factor for SOC and nutrient depletion within Brazilian pastures. Conversion from native vegetation to pasture also degraded soil physical properties. Continuous cattle trampling coupled with SOC depletion, increased soil compaction (i.e., higher BD and SDC) and altered pore size and distribution (i.e., lower MaP and higher MiP). This subsequently reduced soil aeration (SAC), significantly decreased K_fs_ and available water, and may restrict root growth (i.e., higher SRP and VESS scores). Despite those changes, soil aggregate stability (AGS and MWD) was not affected by pasture establishment. Soil compaction and consequently physical degradation of pasturelands are well documented in the literature (e.g., [48–50]).

**Table 3:** Mean values of the 38 soil indicators (0–30 cm depth) in native vegetation (NV), pasture (PA) and sugarcane (SC) at three sites in central-southern Brazil.

	Lat_17S			Lat_21S			Lat_23S		
Indicator^§^	NV	PA	SC	NV	PA	SC	NV	PA	SC
**Chemical**									
P (mg dm^-3^)	4.5 b*	2.6 c	6.7 a	12.9 a	5.1 b	9.8 a	14.5 a	10.9 ab	7.6 b
S (mg dm^-3^)	4.1 b	3.6 b	17.3 a	8.6 a	9.1 a	7.7 a	16.4 a	10.6 b	6.0 b
K (mmol_c_ dm^-3^)	0.8 a	0.5 b	0.4 b	2.7 a	3.1 a	2.5 a	3.0 b	4.4 a	2.0 b
Ca (mmol_c_ dm^-3^)	3.0 b	2.7 b	20.0 a	69.4 a	7.1 c	29.1 b	19.1 b	31.1 b	49.8 a
Mg (mmol_c_ dm^-3^)	2.4 b	1.3 b	8.7 a	17.6 a	4.1 c	13.0 b	9.9 b	17.8 ab	19.6 a
B (mg dm^-3^)	0.2 a	0.1 b	0.1 b	0.5 a	0.2 c	0.4 b	0.6 a	0.3 b	0.3 b
Cu (mg dm^-3^)	3.1 a	0.7 b	3.2 a	0.8 b	1.2 a	1.0 b	1.6 b	2.3 a	1.2 c
Fe (mg dm^-3^)	43.6 b	85.6 a	20.8 b	15.0 c	164.8 a	51.4 b	87.5 a	90.3 a	21.6 b
Mn (mg dm^-3^)	9.7 a	3.6 b	4.9 b	32.6 a	14.3 b	16.5 b	45.5 b	100.5 a	14.7 c
Zn (mg dm^-3^)	0.5 a	0.3 a	0.4 a	2.2 a	1.3 b	1.4 b	2.4 b	4.1 a	0.8 b
CEC_pH7_ (mmol_c_ dm^-3^)	78.6 a	54.3 b	60.3 b	104.6 a	60.9 c	71.0 b	169.5 a	103.0 b	105.2 b
H+Al (mmol_c_ dm^-3^)	72.4 a	49.7 b	31.2 c	14.9 c	46.6 a	26.5 b	137.6 a	49.8 b	33.8 b
pH_CaCl2_ (unitless)	3.7 b	3.7 b	5.0 a	6.1 a	3.9 c	5.0 b	3.8 c	4.6 b	5.4 a
BS (%)	7.9 b	8.6 b	48.2 a	85.5 a	23.6 c	62.1 b	19.6 b	51.5 a	67.1 a
**Physical**									
BD (Mg m^-3^)	1.3 c	1.6 a	1.5 b	1.3 b	1.6 a	1.7 a	1.0 b	1.3 a	1.4 a
SDC (%)	73.8 b	87.7 a	89.8 a	70.9 b	89.3 a	89.3 a	79.6 b	95.4 a	98.3 a
SRP (MPa)	1.1 c	1.9 a	1.5 b	0.6 c	2.8 a	1.9 b	2.4 a	2.4 a	2.2 a
MaP (m^3^ m^-3^)	0.26 a	0.16 b	0.12 b	0.22 a	0.06 b	0.05 b	0.21 a	0.03 b	0.05 b
MiP (m^3^ m^-3^)	0.29 b	0.23 c	0.34 a	0.29 b	0.32 a	0.32 a	0.40 c	0.48 a	0.44 b
TP (m^3^ m^-3^)	0.55 a	0.39 c	0.46 b	0.51 a	0.39 b	0.38 b	0.61 a	0.51 b	0.49 b
WFPS (unitless)	0.40 b	0.37 b	0.62 a	0.37 b	0.54 a	0.63 a	0.48 b	0.87 a	0.81 a
SWSC (unitless)	0.47 b	0.49 b	0.69 a	0.41 b	0.71 a	0.72 a	0.61 b	0.93 a	0.88 a
SAC (unitless)	0.53 a	0.51 a	0.31 b	0.59 a	0.29 b	0.28 b	0.39 a	0.07 b	0.12 b
K_fs_ (cm h^-1^)	130 b	48 b	358 a	129 a	3 b	4 b	46.9 a	1.7 b	0.9 b
AGS (%)	90.0 a	92.7 a	79.2 b	80.5 a	84.5 a	66.7 b	93.7 b	96.7 a	87.0 c
MWD (mm)	3.3 b	4.0 a	1.4 c	4.4 a	4.2 a	3.4 b	4.1 b	4.7 a	2.6 c
VESS (score)	1.8 b	2.0 b	2.5 a	1.8 c	2.9 b	3.7 a	2.5 b	3.2 a	3.3 a
SSI (%)	5.7 b	9.1 a	4.6 c	11.2 a	7.2 b	6.9 b	7.4 a	6.6 b	4.5 c
**Biological**									
SOC (g kg^-1^)	13.1 a	8.8 c	11.0 b	16.3 a	10.2 b	9.4 b	35.5 a	30.5 b	19.5 c
TN (g kg^-1^)	1.0 a	0.5 b	0.9 a	1.7 a	0.9 b	1.0 b	3.1 a	2.3 b	1.5 c
MBC (mg kg^-1^)	421.9 a	396.0 a	375.6 a	841.2 a	450.1 b	559.3 b	2049.5a	2238.2 a	1024.3 b
MBN (mg kg^-1^)	41.0 a	22.6 b	17.0 b	75.7 a	30.1 b	21.7 b	98.4 b	161.9 a	43.3 c
BG (mg kg^-1^ h^-1^)	50.5 a	39.8 a	47.1 a	108.2 c	270.0 a	206.2 b	384.2 a	120.8 b	53.4 b
AcP (mg kg^-1^ h^-1^)	204.5 a	154.2 b	138.2 b	151.6 b	256.2 a	229.4 a	324.3 a	326.2 a	167.8 b
Eworm (indiv m^-2^)	8 a	4 a	4 a	20 b	248 a	36 b	12 b	60 a	4 b
MDens (indiv m^-2^)	120 b	1428 a	40 b	664 a	772 a	148 b	516 a	888 a	72 b
MRich (unitless)	0.4 a	0.2 a	0.3 a	0.9 a	0.5 a	0.4 a	0.6 a	0.4 ab	0.2 b
MDiver (unitless)	0.8 a	0.3 b	0.6 a	1.2 a	1.1 a	0.8 a	1.1 a	0.7 b	0.5 b

*Mean values within each site followed by the same letter do not differ among themselves according to Tukey’s test (*p*<0.05).

^§^Abbreviations are same as Table 1.

Soil biological changes were also observed due to conversion from native vegetation to pasture. Most biological indicators showed site-specific responses, although MBC and MBN tended to be lower within pasture soils, especially at the Lat_17S and Lat_21S sites. Enzyme activities (BG and AcP) showed a decreasing trend under pasture at Lat_17S and Lat_23S, but increased significantly at Lat_21S. Variation in soil acidity, SOC, P availability, microbiological activity and other variables not assessed in this study, may be among the controlling factors affecting enzyme responses at the various sites. Pastures soils generally had a higher density of macrofauna than native vegetation sites, but the increase was dominated by a few taxonomic groups such termites (mainly at Lat_17S), ants, coleopterans and earthworms. Conversely, even though native vegetation samples had lower macrofauna populations, they had a higher richness and diversity of species. Our findings are consistent with others in the literature [51, 52], which generally state that macrofaunal community size in tropical soils tends to increase over time following conversion from native vegetation to pasture.

The LUC from pasture to sugarcane improved soil chemical quality. Liming and annual application of fertilizer (organic and/or mineral) reduced soil acidity and increased macronutrient availability [34]. Short-term sugarcane establishment (<5 years) had no negative impacts on SOC or TN content at Lat_17S and Lat_21S, but as reported by Mello et al. [53] and Franco et al. [54], SOC and TN were depleted after more than 20 years (Lat_23S) of sugarcane cultivation. Those decreases presumably are associated with the intensive tillage performed every five years [53, 55] and more than 10 years of pre-harvest burning, which has been shown to deplete SOC over time [56].

Conversion from pasture to sugarcane also negatively impacted on soil physical indicators, primarily those related to soil structure, such as AGS, MWD, VESS and SSI. Although tillage in preparation for sugarcane replanting (Lat_17S) alleviated soil compaction (i.e., decreased BD and SRP; increased K_fs_), our data suggest those positive effects have short-term persistence (i.e., primarily the first year, as reported by Centurion et al. [57]). Over the entire sugarcane cycle, intensive machinery traffic increases soil compaction again, leading to decreased of aeration, infiltration and water availability as observed at Lat_21S and Lat_23S. Short-term positive tillage effects on soil physical quality are most likely associated with SOC depletion, due to disruption of macroaggregates and exposure of physically and chemically protected C to microbial decomposition [58] and the subsequent deleterious consequences on soil structure. In addition, several studies have shown that intensive machinery traffic in sugarcane fields has negative impacts on soil physical quality and often decreases sugarcane growth and yield (e.g., [59, 60]). Adverse impacts of current sugarcane management practices on soil physical and structural properties have also markedly increased soil loss and degradation by erosion when compared with native vegetation or pasture [61]. Degraded SQ has thus become a major concern for a sustainable sugarcane production in Brazil [62].

Overall, LUC from pasture to sugarcane also has negative implications on soil biological indicators. Depletions of soil biota in sugarcane fields can be associated with quantitative and qualitative decreases in SOC. Franco et al. [54] reported that sugarcane production depletes C input from C_3_ plants (forest) which is preferable by microorganisms, and that new C from C_4_ plants (i.e., pasture and sugarcane) was insufficient to offset those losses. Furthermore, intensification of land use and management, that includes considerable mineral fertilizer and pesticides inputs as well as the modification or destruction of native biological habitats by tillage, and soil compaction can led to a reduction or simplification in soil diversity and its ecosystem functions in sugarcane fields [63].

### Soil quality indexing

The three SQ indicator selection approaches (Fig 2) provided different datasets for index calculations. The TDS (38 indicators) provided a wide range of soil indicators and theoretically should have resulted in a more accurate (sensitive) assessment of SQ, due to the very comprehensive evaluation involving chemical, physical and biological soil properties and their interactions. The primary limitations of the TDS approach are the high cost, greater amount of time required for sampling and laboratory analyses, redundancy of indicators, and more complex data interpretation [18, 21, 26]. Using a PCA reduced the TDS to seven principal components (MDS_-PCA_) that explained approximately 90% of total variance (Fig 3 and Table 4). The seven indicators were: SOC, SAC, pH, K_fs,_ Mdiver, BG and Mdens. Selecting SQ indicators using PCA has some advantages and disadvantages. According to Andrews et al. [12] and Mukherjee and Lal [9], PCA provides a less subjective method of indicator selection, which can help avoid bias and data redundancy. On the other hand, the PCA method requires a large dataset and is less “user friendly,” thus imposing barriers to practical adoption for farm or regional scale SQ assessments. Furthermore, the selected indicators may not be meaningful for farmers and land managers [12]. The expert opinion approach reduced the TDS to five MDS_-EO_ indicators (pH, P, K, VESS and SOC), with the first three being chemical indicators that are widely used to evaluate soil acidity and nutrient availability as well as to guide soil fertility management. As recommended by Doran and Parkin [25], these indicators are desirable for SQ assessments because they are: easy to sample for, readily available in commercial laboratories at a low cost, and the results can be easily interpreted using pre-defined thresholds. The fourth indicator, VESS score, provides an integrative assessment of soil structural/physical quality through an easily-performed, low-cost, direct on-farm method [37, 38]. VESS integrates soil properties related to size, strength and porosity of aggregates, roots and soil color into a single score, that ranges from 1 (good) to 5 (poor structural quality) [37, 38]. The fifth indicator, SOC, is the most consistent indicator used for SQ assessments [7] because it influences multiple soil and ecosystem functions [64]. Furthermore, SOC can be analyzed using the same sample collected for chemical indicators and it is routinely analyzed so most farmers have previous records for temporal comparisons. The MDS_-EO_ approach was consistent with Andrews et al. [10] and Karlen et al. [65], who recommend that SQ assessments could be made using a minimum of five indicators provided there was at least one each representing soil chemical, physical and biological properties and processes. However, Andrews et al. [12] did warn that the expert opinion method does truly require expert knowledge of the entire production system and may be subject to disciplinary bias.

**Table 4:** Result of principal component analysis.

	Principal Components							
	PC1	PC2	PC3	PC4	PC5	PC6	PC7	
Eigenvalues	10.33	7.92	5.76	3.17	2.53	2.31	2.19	
Variance (%)	27.19	20.84	15.15	8.35	6.67	6.08	5.75	
Cumulative (%)	27.19	48.03	63.18	71.53	78.20	84.27	90.03	
**Soil Indicators**	**Eigenvectors**^‡^							Communalities
P	0.685	-0.035	0.482	-0.018	0.122	0.421	0.076	0.901
S	0.319	0.166	0.009	0.656	0.147	0.508	0.179	0.871
K	0.553	0.395	0.194	-0.369	0.424	0.061	0.198	0.859
Ca	0.243	-0.008	**0.930**	-0.132	0.035	0.044	-0.049	0.947
Mg	0.407	0.343	0.797	-0.086	-0.037	0.027	-0.066	0.933
B	0.610	-0.216	0.387	-0.248	0.130	0.517	-0.094	0.922
Cu	0.170	0.047	-0.251	0.799	0.023	-0.328	-0.222	0.890
Fe	0.030	0.257	-0.660	-0.329	0.269	0.106	0.416	0.867
Mn	0.815	0.296	0.111	-0.060	0.204	-0.222	0.285	0.940
Zn	0.736	0.198	0.199	-0.103	0.296	-0.080	0.328	0.833
CEC	0.849	-0.114	0.076	-0.061	0.007	0.418	-0.183	0.951
H+Al	0.580	-0.190	-0.656	0.052	-0.023	0.374	-0.144	0.967
pH	-0.046	0.063	**0.981**	0.048	0.032	-0.028	-0.053	0.976
BS	0.042	0.202	**0.966**	-0.028	0.099	0.031	-0.027	0.987
BD	-0.832	0.375	-0.009	-0.167	-0.049	-0.065	0.286	0.950
SDC	-0.131	**0.900**	-0.008	-0.040	-0.237	-0.080	0.054	0.895
RP	0.123	0.727	-0.408	-0.244	-0.051	0.213	0.158	0.843
MaP	0.187	**-0.932**	-0.147	0.144	-0.024	0.025	-0.179	0.980
MiP	0.641	0.702	0.163	0.114	0.029	0.002	-0.143	0.964
TP	0.817	-0.326	-0.016	0.249	0.019	0.035	-0.350	0.960
WFPS	0.215	**0.899**	0.230	0.108	-0.022	-0.156	-0.097	0.954
SWSC	0.184	**0.964**	0.073	0.051	-0.031	-0.037	-0.041	0.975
SAC	-0.184	**-0.964**	-0.073	-0.051	0.031	0.037	0.041	0.975
K_fs_	-0.214	-0.302	0.141	**0.858**	-0.037	-0.067	-0.040	0.901
AGS	0.607	-0.060	-0.477	-0.080	-0.235	-0.388	0.085	0.820
MWD	0.448	-0.150	-0.097	-0.667	0.309	-0.119	0.383	0.934
VESS	-0.011	0.832	0.111	-0.115	-0.039	0.322	0.010	0.823
SSI	0.082	-0.586	0.227	-0.477	0.170	0.061	0.511	0.922
SOC	**0.963**	0.150	-0.031	0.001	0.016	0.134	-0.052	0.972
TN	**0.929**	0.063	0.055	-0.032	0.114	0.277	-0.056	0.963
MBC	**0.903**	0.279	0.019	-0.087	-0.002	0.086	0.085	0.916
MBN	0.866	0.160	0.082	-0.089	0.086	-0.209	0.202	0.883
BG	0.367	0.099	-0.274	-0.210	0.342	**0.729**	0.125	0.928
AcP	0.629	0.340	-0.402	-0.169	0.391	0.172	0.072	0.890
Eworm	-0.163	0.215	-0.235	-0.003	0.322	0.128	0.520	0.518
Mdens	0.065	-0.136	-0.134	-0.156	-0.237	-0.013	**0.746**	0.678
Mrich	0.236	-0.396	0.186	-0.075	0.749	0.079	0.034	0.822
Mdiver	0.131	-0.184	-0.020	0.015	**0.909**	0.129	-0.089	0.903

^**‡**^Bold values under each component were highly weighted (factor loading value within 10% of the highest values under the same principal component) and underlined bold values were selected to minimum dataset.

^§^Abbreviations are same as Table 1.

**Fig 5:**
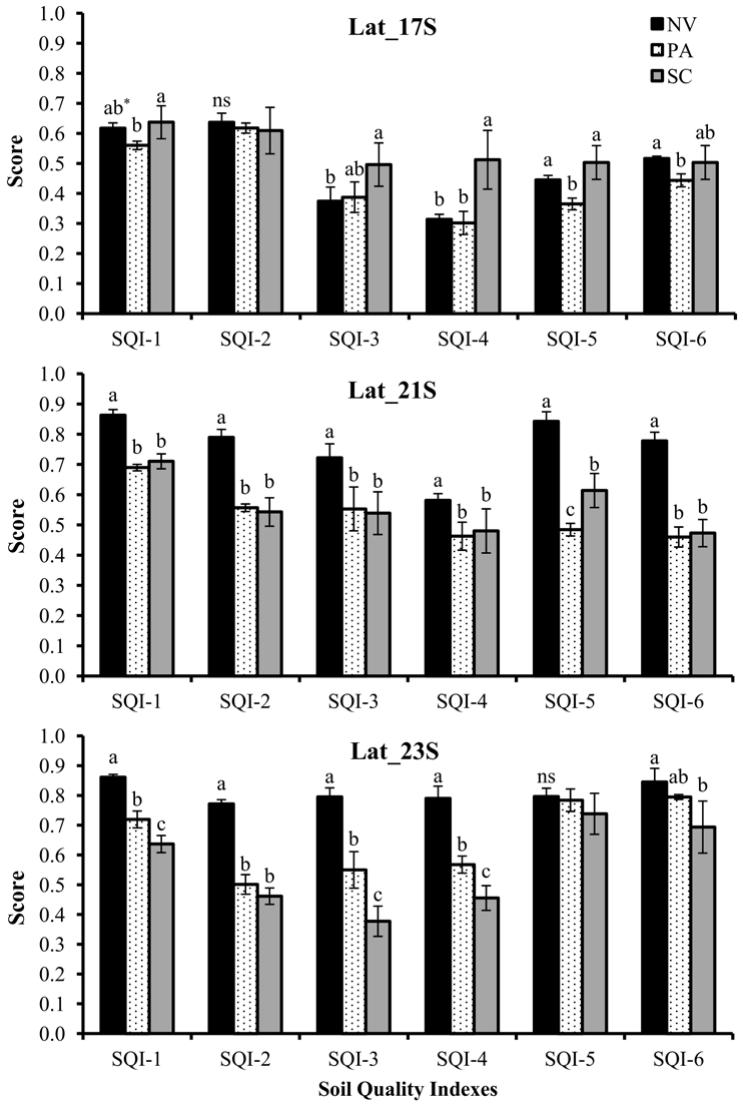
Soil Quality Index (SQI)^§^ scores under native vegetation (NV), pasture (PA) and sugarcane (SC), for the 0–30 cm depth, at three sites in central-southern Brazil. ^§^SQI strategies: SQI-1: TDS/non-linear/simple additive, SQI-2: TDS/non-linear/weighted additive, SQI-3: MDS_-PCA_/non-linear/simple additive, SQI-4: MDS_-PCA_/non-linear/ weighted additive, SQI-5: MDS_-EO_ /non-linear/simple additive, and SQI-6: MDS_-EO_ /non-linear/weighted additive. *Mean values within each index followed by the same letter do not differ among themselves according to Tukey’s test (*p*<0.05).

Soil quality indicators were individually scored (Eqs 1 and 2) and then, integrated using six strategies (Fig 2). The SQI scores for native vegetation, pasture and sugarcane (0–30 cm depth) at each site are shown in Fig 5. Overall, all six SQI approaches were able to detect SQ changes induced by LUC. Soils from native vegetation sites had significantly greater SQI values, except at Lat_17S, where the soil was more weathered and consequently had very poor chemical quality [34]. In general, LUC from native vegetation to pasture significantly decreased SQ, although the sensitivity among the SQ indexing strategies was slightly different. Conversion from pasture to sugarcane promoted site-specific SQ changes, leading to increases or decreases associated with inherent soil characteristics and historic of land use and management. At Lat_17S, sugarcane cultivation increased SQ, primarily due to soil fertility improvement through lime and fertilizer applications. This was confirmed by SQIs calculated using strategies that gave greater weight to chemical indicators, such as SQI-4 and SQI-5. At Lat_21S, conversion from pasture to sugarcane had essentially no influence on overall SQ, except when SQI-5 was used for the evaluation. In contrast, at Lat_23S, SQI-1, SQI-3 and SQI-4 indicated that long-term sugarcane cultivation significantly decreased SQ likely due to significant SOM depletion [54]. This in turn had negative implications on micro- and macro-faunal activity, cycling and availability of nutrients, and soil structure (Table 3).

**Fig 6:**
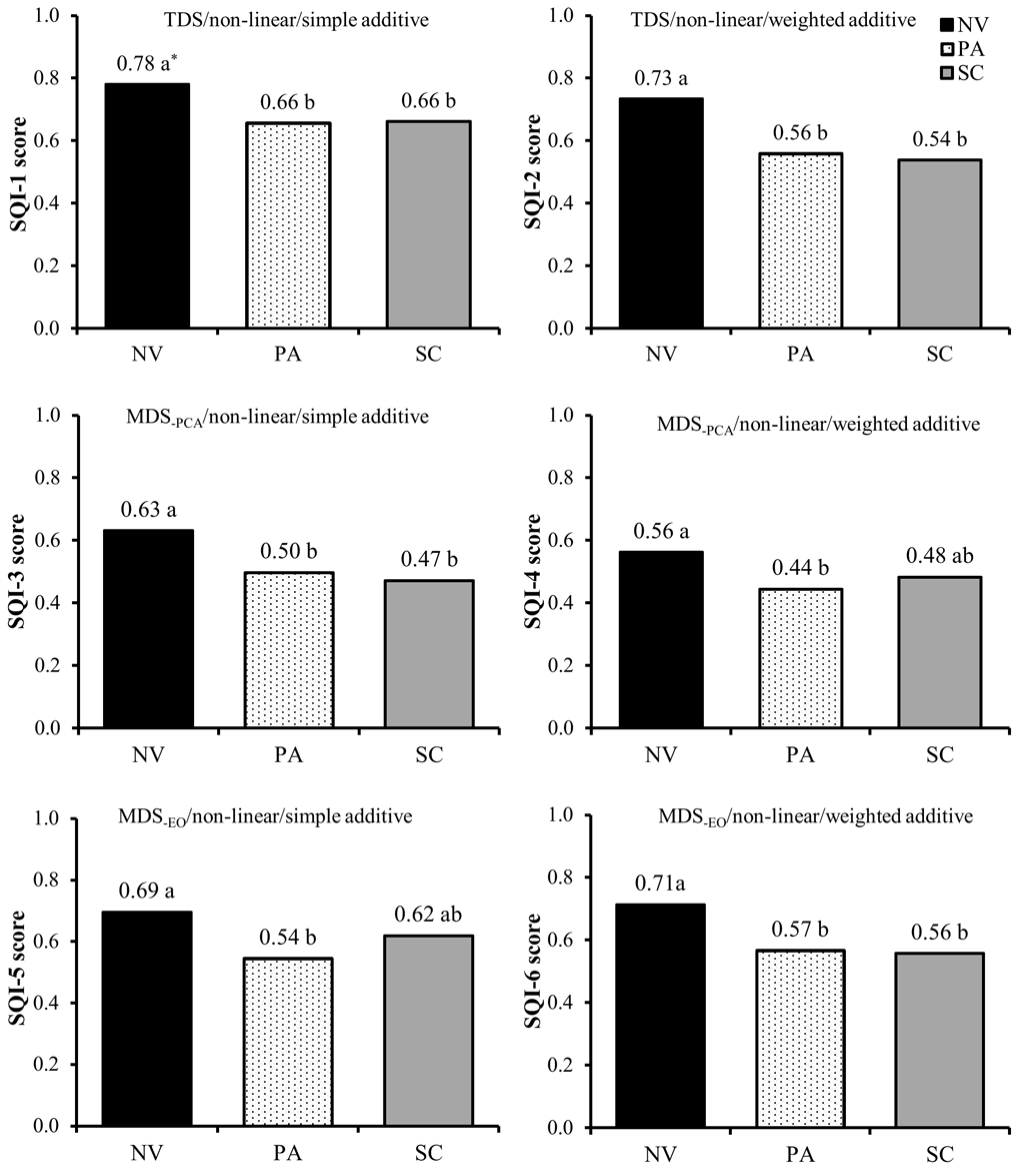
Overall Soil Quality Index (SQI) scores under native vegetation (NV), pasture (PA) and sugarcane (SC), for the 0–30 cm depth, in central-southern Brazil. *Mean values within each index followed by the same letter do not differ among themselves according to Tukey’s test (*p*<0.05).

**Fig 7:**
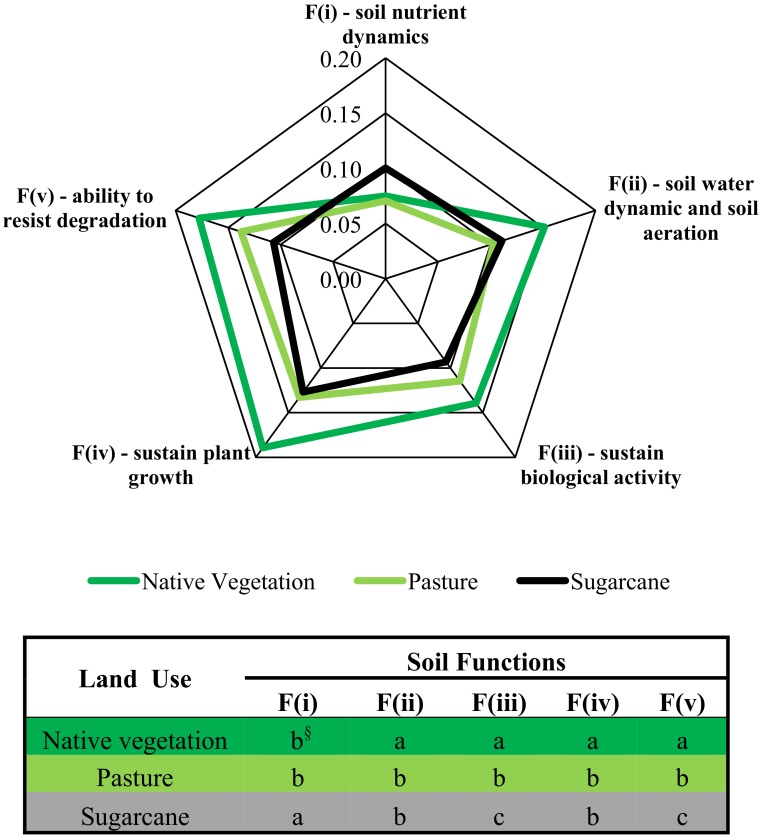
Contribution of each soil functions in the SQI-2 under native vegetation, pasture and sugarcane in central-southern Brazil. ^§^Same letter within each soil function indicates that the mean values do not differ among land uses according to Tukey’s test (*p*<0.05).

**Fig 8:**
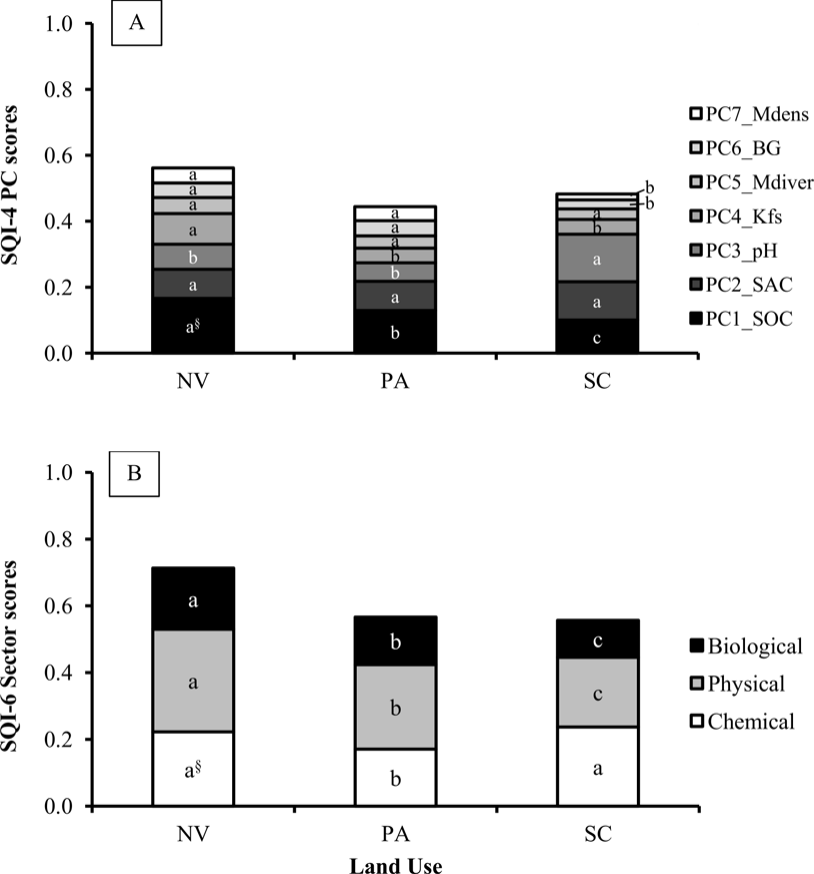
**Contribution of each principal component (PC) and soil sector in the SQI-4 (A) and SQI-6 (B), respectively under native vegetation (NV), pasture (PA) and sugarcane (SC) in central-southern Brazil.** ^§^Same letter within each PC or soil sector indicates that the mean values do not differ among land uses according to Tukey’s test (*p*<0.05).

At the regional scale, SQ changes induced by LUC were consistently detected by all six indexing strategies (Fig 6). In general, higher absolute SQI values were observed when using the TDS (SQI-1 and SQI-2) followed by the SQIs from MDS_-EO_ (SQI-5 and SQI-6) and SQIs from MDS_-PCA_ (SQI-3 and SQI-4). An identical sequence was verified by Lima et al. [26]. Native vegetation soils had the highest SQI scores, suggesting they are functioning at 56 to 78% of their potential capacity for the 0–30 cm depth. These results support the hypothesis that natural ecosystems are more balanced, because chemical, physical and biological attributes act collectively, thus enabling soils to perform their functions properly. The SQIs indicated that long-term conversion from native vegetation to extensive pasture decreased SQ indexes by 15 to 23% (Fig 6), resulting in pasture soils that were functioning at between 44 to 66% of their potential capacity. Weighed indexes helped clarify the reasons for overall SQ depletion within pasturelands. The SQI-2 scores (i.e., TDS weighted by soil function framework) indicated that pasture soils had reduced soil functions associated with storage and provision of water, as well as soil aeration (-32%), soil capacity to sustain plant growth (-18%), biological activity (-30%), ability to resist degradation (-22%), and although not statistically significant, the capacity for storage, provision and cycling of nutrients (-6%) when compared to soils under native vegetation (Fig 7). The SQI-4, weighted by PCA loading, showed that SQ depletions in pasturelands were mainly associated with significant decreases of SOC and K_fs_ (Fig 8A). Using only five selected, but weighted soil indicators (SQI-6) detected that SQ depletion due to conversion from native vegetation to pasture was associated with significant decreases in soil chemical (-23%), physical (-17%) and biological sectors (-22%) (Fig 8B). This was in agreement with results obtained using SQI-2, which was the most complex strategy.

Our results effectively described the critical situation associated with most Brazilian pastureland. It is estimated that 70% of those areas are degraded or in the process of being degraded [4]. Recently, a national-scale study verified that the current productivity (i.e., animal unit carrying capacity) of cultivated pasturelands is only 32–34% of their inherent potential [5]. The low productivity of Brazilian pasturelands has multiple causes as reported by Strassburg et al. [5]. Among them are improper pasture management, including seedling failures and bare soil, continuous grazing, absence of liming, maintenance of soil fertility through fertilization, and uncontrolled erosion, which all lead to soil degradation over time [4, 5].

At the regional scale, LUC from pasture to sugarcane showed no significant impact on overall SQ (Fig 6). The SQI scores suggest that sugarcane soils are functioning at 47 to 66% of their capacity. The SQI strategies showed sparse non-significant variations on SQ under sugarcane compared to pasture, ranging from -6% (SQI-3) to +13% (SQI-5). Respectively, SQI-3 and SQI-5 were the indexes that gave the lowest and the highest weight to chemical indicators. Therefore, improving soil fertility attenuated negative implications of sugarcane production on soil physical and biological indicators within overall SQ assessment. This was clearly demonstrated by weighted indexes (SQI-2, SQI-4 and SQI-6). Conversion from pasture to sugarcane had one positive effect on soil functions–that related to nutrient dynamics. In contrast, significant adverse effects were observed in soil functions related to the capacities to sustain biological activity and resist to degradation (Fig 7). SQI-4 showed that under sugarcane only pH scores was improved, while SOC, BG and Mdens scores decreased (Fig 8A). Finally, SQI-6 also was able to indicate that sugarcane production led to significant improvement on soil chemical indicators and decline on physical and biological indicators. Overall, these results indicate that sugarcane expansion over degraded pasturelands seems to be an opportune way to meet increasing domestic and global ethanol demands, avoiding direct competition for land with food crops and natural ecosystems, as reported by Goldemberg et al. [2] and Strassburg et al. [5]. However, the results clearly indicated the necessity for improved management practices that can mitigate deleterious impacts of sugarcane production on soil physical/structural and biological indicators.

#### What is the best indexing strategy for assessing sugarcane expansion impacts on soil quality?

**Fig 9:**
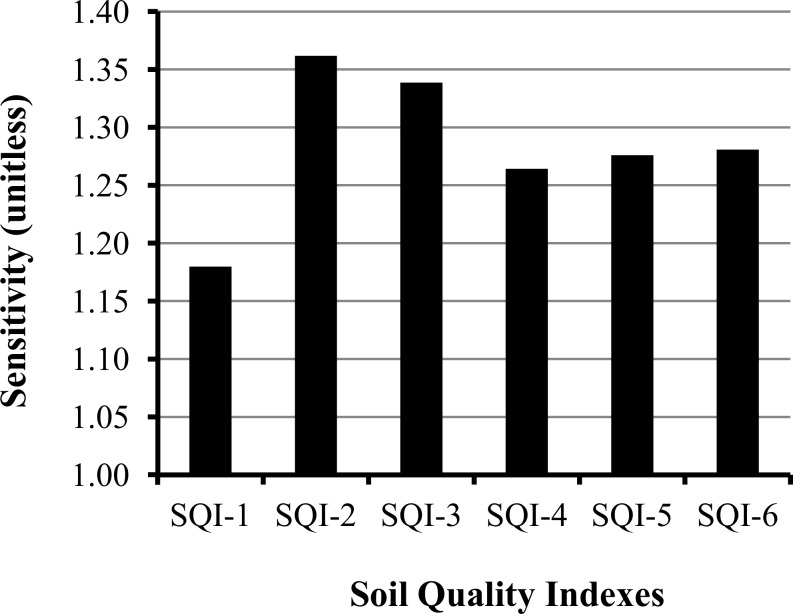
Sensitivity values of SQ indexing strategies used to assess the land use change (native vegetation—pasture—sugarcane) impacts on soil quality in central-southern Brazil.

All six SQ indexing strategies were able to detect SQ changes induced by LUC, suggesting that any of them could be used for monitoring SQ in sugarcane expansion in Brazil (Fig 6). However, a sensitivity test showed there were slight differences among the strategies (Fig 9). The most complex strategy (SQI-2), which included the 38 indicator TDS and used weighting of the indicator scores provided by the soil function framework had greatest sensitivity to detect SQ changes due to LUC. In contrast, the least sensitive SQI was calculated using TDS without indicator weighting (SQI-1). These results suggest that using a meaningful method (e.g., soil functions) for weighting and integrating indicator scores into an index when a large dataset is available for SQ assessment is best, even though it is more complex than simple additive indexing and does not statistically modify the overall SQ assessment response (Fig 6). There also is no consensus in the literature regarding the benefits of indicator weighting. Andrews et al. [12] and Askari and Holden [18] concluded that weighting an additive SQI did not change the relative rankings among treatments, and therefore, this extra step was unnecessary for analyzing vegetable production or other systems. Mukherjee and Lal [9] also reported similar effectiveness between simple and weighted indexes. They highlighted that appropriate weighting on scores can predict SQ with higher performance which was consistent with our findings. On the other hand, Askari and Holden [19] showed that a simple additive linear SQI was the most efficient for detecting management practice impacts in arable soils.

Both the MDS_-PCA_ (SQI-3 and SQI-4) and MDS_-EO_ (SQI-5 and SQI-6) strategies were effective for detecting SQ changes using a reduced number of indicators (Fig 9). Similar results were reported by Andrews et al. [12] and Lima et al. [26], who both concluded that a reduced number of carefully chosen indicators could adequately provide the information needed for decision-making. From a practical perspective, this means that for any one indexing strategy to become the standard for research, large-scale SQ assessments, or to facilitate discussion and cooperation, it must be rapid, reliable, and economically feasible [21]. SQI-5 and SQI-6 strategies have an important advantage compared to SQI-3 and SQI-4, since the latter two require a large dataset in order to perform a PCA and select fewer indicators. Therefore, simple SQI strategies (SQI-5 and SQI-6), show excellent potential for monitoring SQ changes in sugarcane expansion areas in Brazil. More specifically, between SQI-5 and SQI-6, we suggest opting for SQI-6, which provides balanced weighting for chemical, physical and biological indicators.

**Fig 10:**
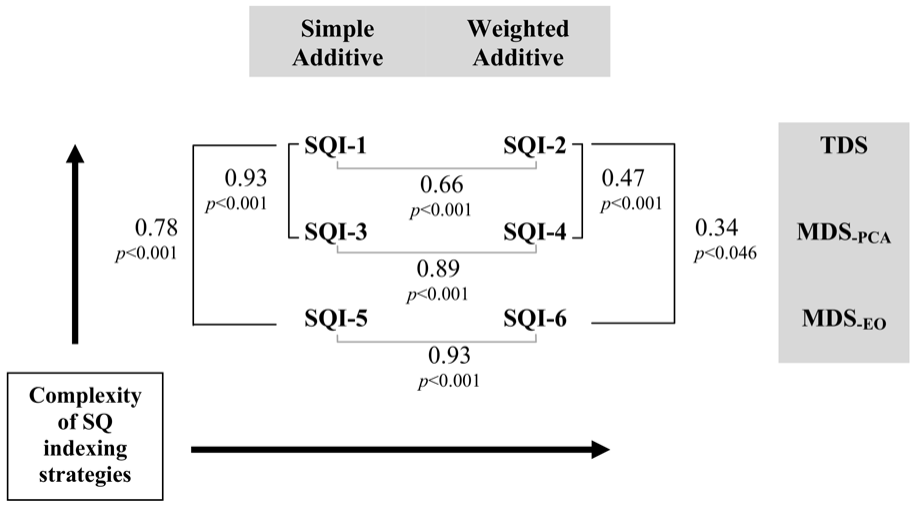
Pearson’s correlation coefficients and probability of error (*p*) among soil quality indexes (SQI) developed to assess the land use change (native vegetation—pasture—sugarcane) impacts on soil quality in central-southern Brazil.

The SQ indexing strategies were significantly correlated among themselves (Fig 10), confirming a close relationship between simple and more complex strategies. This was also reported by Mukherjee and Lal [9]. Decreasing correlations between SQI-1 *vs* SQI-3 (r = 0.93), SQI-1 *vs* SQI-5 (r = 0.78), SQI-2 *vs* SQI-4 (r = 0.47), and SQI-2 *vs* SQI-6 (r = 0.34) were observed. This indicates that as the indexes became simpler, correlations with more complex indexes that used the entire dataset were lower. However, despite lower correlations, simple indexing strategies (i.e., SQI-5 and SQI-6) had the same statistical ability for ranking SQ responses due to LUC (Fig 6) as the more complex strategies.

Simple additive indexes had greater correlations among themselves than weighted indexes (Fig 10), because the simple ones were calculated using only a different number of indicators, while weighted indexes also varied the weighting approaches. Higher correlations were verified between simple additive and weighted additive indexes when fewer indicators were selected (i.e., SQI-1 *vs* SQI-2, r = 0.66; SQI-3 *vs* SQI-4, r = 0.89; SQI-5 *vs* SQI-6, r = 0.92). Fig 9 confirms there was a decreasing trend for sensitivity differences between simple and weighted indexes derived from the same sequence, as the comparisons moved from more complex to simpler strategies.

## Conclusions

All six indexing strategies efficiently detected SQ changes due to LUC for sugarcane expansion in Brazilian tropical soils. Both, PCA and EO approaches were useful to reduce the total dataset without any significant interference on SQ ranking among land uses. These results indicate that simple, easily-performed and more user-friendly SQI strategies (e.g., SQI-5 and SQI-6) were as effective and suitable for detecting LUC effects on SQ as more complex SQI strategies (e.g., SQI-1, SQI-2, SQI-3 and SQI-4). Although simple additive and weighted additive SQIs were statistically similar, we recommend using weighted indexes, especially when the number of indicators is unbalanced among chemical, physical and biological components. Therefore, a SQI strategy using a small number of carefully chosen soil indicators, such as pH, P, K, VESS and SOC, and proportional weighting for indicator scores within of each soil sector (chemical, physical and biological) could be adopted as a protocol for SQ assessments in Brazilian sugarcane areas.

Our findings also suggest that long-term LUC from native vegetation to extensive pasture depleted overall SQ, driven by decreases in chemical, physical and biological indicators. In contrast, conversion from pasture to sugarcane had no significant impact on overall SQ, primarily, because chemical improvements offset negative impacts on biological and physical indicators. Therefore, sugarcane expansion into degraded pastureland seems to be a sustainable strategy to meet increasing demands for biofuels. Nevertheless, management practices that alleviate soil physical and biological degradation under sugarcane production must be prioritized to avoid or minimize SQ depletions over time.

## Supporting Information

S1 Table Pearson’s correlation coefficients (r) among soil chemical, physical and biological indicators in the land use change areas in central-southern Brazil. (DOCX)

S2 Table Model of soil functions framework and indicators used to develop the SQI-2. (DOCX)
