# Supplementary material for: Soil Quality Indexing Strategies for Evaluating Sugarcane Expansion in Brazil
Source: PLoS One. 2016 Mar 3;11(3):e0150860. doi: 10.1371/journal.pone.0150860 (PMC4777567; doi:10.1371/journal.pone.0150860)
Supplement: S1 Table — (DOCX) [file pone.0150860.s001.docx]

**Electronic supporting information**

**S1 Table.** Pearson’s correlation coefficients (r) among soil chemical, physical and biological indicators^§^ in the land use change areas in central-southern Brazil.

|  | **S** | **K** | **Ca** | **Mg** | **B** | **Cu** | **Fe** | **Mn** | **Zn** | **CEC** | **pH** | **BS** | **H+Al** | | **BD** | | **SDC** | | **RP** | | **MaP** | | **MiP** | | **TP** | |
| --- | --- | --- | --- | --- | --- | --- | --- | --- | --- | --- | --- | --- | --- | --- | --- | --- | --- | --- | --- | --- | --- | --- | --- | --- | --- | --- |
| **P** | 0.41 | 0.60 | 0.61 | 0.65 | 0.83 | -0.12 | -0.18 | 0.56 | 0.65 | 0.76 | 0.41 | 0.51 | 0.22 | -0.58 | | -0.19 | | -0.05 | | 0.08 | | 0.47 | | 0.53 | |  |
| **S** | 1.00 | 0.13 | 0.02 | 0.11 | 0.24 | 0.32 | 0.02 | 0.25 | 0.26 | 0.40 | 0.02 | 0.06 | 0.35 | -0.27 | | 0.05 | | 0.13 | | -0.05 | | 0.36 | | 0.30 | |  |
| **K** |  | 1.00 | 0.35 | 0.53 | 0.46 | -0.23 | 0.35 | 0.72 | 0.72 | 0.42 | 0.17 | 0.34 | 0.06 | -0.23 | | 0.16 | | 0.39 | | -0.38 | | 0.60 | | 0.18 | |  |
| **Ca** |  |  | 1.00 | 0.86 | 0.55 | -0.32 | -0.55 | 0.26 | 0.33 | 0.34 | 0.92 | 0.93 | -0.46 | -0.23 | | -0.05 | | -0.29 | | -0.09 | | 0.32 | | 0.20 | |  |
| **Mg** |  |  |  | 1.00 | 0.50 | -0.18 | -0.43 | 0.48 | 0.47 | 0.42 | 0.79 | 0.86 | -0.33 | -0.25 | | 0.24 | | 0.03 | | -0.35 | | 0.64 | | 0.24 | |  |
| **B** |  |  |  |  | 1.00 | -0.34 | -0.19 | 0.39 | 0.49 | 0.81 | 0.30 | 0.39 | 0.34 | -0.60 | | -0.33 | | -0.14 | | 0.24 | | 0.29 | | 0.54 | |  |
| **Cu** |  |  |  |  |  | 1.00 | -0.19 | 0.11 | -0.01 | -0.06 | -0.20 | -0.25 | 0.18 | -0.31 | | -0.07 | | -0.17 | | 0.19 | | 0.21 | | 0.40 | |  |
| **Fe** |  |  |  |  |  |  | 1.00 | 0.18 | 0.20 | -0.06 | -0.64 | -0.54 | 0.35 | 0.22 | | 0.16 | | 0.60 | | -0.28 | | 0.02 | | -0.26 | |  |
| **Mn** |  |  |  |  |  |  |  | 1.00 | 0.92 | 0.51 | 0.07 | 0.21 | 0.22 | -0.45 | | 0.13 | | 0.23 | | -0.21 | | 0.68 | | 0.42 | |  |
| **Zn** |  |  |  |  |  |  |  |  | 1.00 | 0.53 | 0.16 | 0.28 | 0.19 | -0.42 | | 0.01 | | 0.15 | | -0.17 | | 0.57 | | 0.37 | |  |
| **CEC** |  |  |  |  |  |  |  |  |  | 1.00 | 0.03 | 0.10 | 0.67 | -0.84 | | -0.24 | | 0.07 | | 0.28 | | 0.52 | | 0.81 | |  |
| **pH** |  |  |  |  |  |  |  |  |  |  | 1.00 | 0.97 | -0.69 | 0.02 | | 0.04 | | -0.36 | | -0.19 | | 0.20 | | -0.03 | |  |
| **BS** |  |  |  |  |  |  |  |  |  |  |  | 1.00 | -0.64 | 0.02 | | 0.13 | | -0.23 | | -0.32 | | 0.33 | | -0.03 | |  |
| **H+Al** |  |  |  |  |  |  |  |  |  |  |  |  | 1.00 | -0.62 | | -0.26 | | 0.22 | | 0.42 | | 0.17 | | 0.62 | |  |
| **BD** |  |  |  |  |  |  |  |  |  |  |  |  |  | 1.00 | | 0.52 | | 0.25 | | -0.60 | | -0.36 | | -0.99 | |  |
| **SDC** |  |  |  |  |  |  |  |  |  |  |  |  |  |  | | 1.00 | | 0.67 | | -0.90 | | 0.53 | | -0.47 | |  |
| **RP** |  |  |  |  |  |  |  |  |  |  |  |  |  |  | |  | | 1.00 | | -0.64 | | 0.46 | | -0.24 | |  |
| **MaP** |  |  |  |  |  |  |  |  |  |  |  |  |  |  | |  | |  | | 1.00 | | -0.52 | | 0.57 | |  |
| **MiP** |  |  |  |  |  |  |  |  |  |  |  |  |  |  | |  | |  | |  | | 1.00 | | 0.40 | |  |
| **TP** |  |  |  |  |  |  |  |  |  |  |  |  |  |  | |  | |  | |  | |  | | 1.00 | |  |
| **WFPS** |  |  |  |  |  |  |  |  |  |  |  |  |  |  | |  | |  | |  | |  | |  | |  |
| **SWSC** |  |  |  |  |  |  |  |  |  |  |  |  |  |  | |  | |  | |  | |  | |  | |  |
| **SAC** |  |  |  |  |  |  |  |  |  |  |  |  |  |  | |  | |  | |  | |  | |  | |  |
| **K_fs_** |  |  |  |  |  |  |  |  |  |  |  |  |  |  | |  | |  | |  | |  | |  | |  |
| **AGS** |  |  |  |  |  |  |  |  |  |  |  |  |  |  | |  | |  | |  | |  | |  | |  |
| **MWD** |  |  |  |  |  |  |  |  |  |  |  |  |  |  | |  | |  | |  | |  | |  | |  |
| **VESS** |  |  |  |  |  |  |  |  |  |  |  |  |  |  | |  | |  | |  | |  | |  | |  |
| **SSI** |  |  |  |  |  |  |  |  |  |  |  |  |  |  | |  | |  | |  | |  | |  | |  |
| **SOC** |  |  |  |  |  |  |  |  |  |  |  |  |  |  | |  | |  | |  | |  | |  | |  |
| **TN** |  |  |  |  |  |  |  |  |  |  |  |  |  |  | |  | |  | |  | |  | |  | |  |
| **MBC** |  |  |  |  |  |  |  |  |  |  |  |  |  |  | |  | |  | |  | |  | |  | |  |
| **MBN** |  |  |  |  |  |  |  |  |  |  |  |  |  |  | |  | |  | |  | |  | |  | |  |
| **BG** |  |  |  |  |  |  |  |  |  |  |  |  |  |  | |  | |  | |  | |  | |  | |  |
| **AcP** |  |  |  |  |  |  |  |  |  |  |  |  |  |  | |  | |  | |  | |  | |  | |  |
| **Eworm** |  |  |  |  |  |  |  |  |  |  |  |  |  |  | |  | |  | |  | |  | |  | |  |
| **Mdens** |  |  |  |  |  |  |  |  |  |  |  |  |  |  | |  | |  | |  | |  | |  | |  |
| **Mrich** |  |  |  |  |  |  |  |  |  |  |  |  |  |  | |  | |  | |  | |  | |  | |  |
| **Mdiver** |  |  |  |  |  |  |  |  |  |  |  |  |  |  | |  | |  | |  | |  | |  | |  |

**Continuation …**

|  | **WFPS** | **SWSC** | **SAC** | **K_fs_** | **AGS** | **MWD** | **VESS** | **SSI** | **SOC** | **TN** | **MBC** | **MBN** | **BG** | **AcP** | **Eworm** | **MDens** | **MRich** | **MDiver** |
| --- | --- | --- | --- | --- | --- | --- | --- | --- | --- | --- | --- | --- | --- | --- | --- | --- | --- | --- |
| **P** | 0.15 | 0.09 | -0.09 | -0.10 | -0.03 | 0.30 | 0.16 | 0.27 | 0.69 | 0.78 | 0.66 | 0.57 | 0.51 | 0.37 | -0.16 | -0.01 | 0.37 | 0.21 |
| **S** | 0.19 | 0.22 | -0.22 | 0.42 | -0.05 | -0.23 | 0.21 | -0.18 | 0.40 | 0.44 | 0.35 | 0.19 | 0.42 | 0.27 | 0.06 | -0.08 | 0.15 | 0.21 |
| **K** | 0.45 | 0.44 | -0.44 | -0.53 | 0.11 | 0.66 | 0.40 | 0.19 | 0.57 | 0.60 | 0.66 | 0.65 | 0.52 | 0.66 | 0.13 | 0.06 | 0.35 | 0.30 |
| **Ca** | 0.23 | 0.10 | -0.10 | -0.04 | -0.25 | 0.08 | 0.10 | 0.28 | 0.22 | 0.31 | 0.24 | 0.27 | -0.10 | -0.22 | -0.19 | -0.14 | 0.28 | 0.08 |
| **Mg** | 0.59 | 0.46 | -0.46 | -0.16 | -0.14 | 0.08 | 0.36 | -0.03 | 0.42 | 0.44 | 0.46 | 0.42 | -0.02 | 0.03 | -0.16 | -0.10 | 0.10 | -0.03 |
| **B** | -0.07 | -0.09 | 0.09 | -0.26 | -0.05 | 0.36 | 0.05 | 0.39 | 0.62 | 0.74 | 0.56 | 0.45 | 0.54 | 0.37 | -0.17 | -0.03 | 0.45 | 0.30 |
| **Cu** | 0.17 | 0.10 | -0.10 | 0.60 | 0.17 | -0.45 | -0.14 | -0.61 | 0.12 | 0.03 | 0.03 | 0.11 | -0.25 | 0.07 | -0.10 | -0.27 | -0.17 | -0.05 |
| **Fe** | -0.02 | 0.16 | -0.16 | -0.48 | 0.28 | 0.49 | 0.18 | 0.12 | 0.07 | 0.04 | 0.12 | 0.11 | 0.54 | 0.57 | 0.43 | 0.34 | 0.01 | 0.15 |
| **Mn** | 0.46 | 0.42 | -0.42 | -0.32 | 0.43 | 0.55 | 0.21 | 0.12 | 0.77 | 0.73 | 0.84 | 0.91 | 0.26 | 0.68 | 0.05 | 0.13 | 0.20 | 0.15 |
| **Zn** | 0.35 | 0.30 | -0.30 | -0.33 | 0.25 | 0.58 | 0.16 | 0.26 | 0.70 | 0.70 | 0.71 | 0.79 | 0.34 | 0.63 | 0.10 | 0.11 | 0.28 | 0.23 |
| **CEC** | 0.06 | 0.05 | -0.05 | -0.24 | 0.35 | 0.30 | 0.01 | 0.10 | 0.88 | 0.91 | 0.73 | 0.56 | 0.54 | 0.50 | -0.16 | -0.02 | 0.34 | 0.24 |
| **pH** | 0.27 | 0.13 | -0.13 | 0.17 | -0.48 | -0.17 | 0.12 | 0.13 | -0.06 | 0.02 | -0.02 | 0.05 | -0.29 | -0.42 | -0.19 | -0.22 | 0.14 | 0.00 |
| **BS** | 0.40 | 0.27 | -0.27 | 0.04 | -0.48 | -0.07 | 0.28 | 0.12 | 0.04 | 0.13 | 0.12 | 0.16 | -0.15 | -0.24 | -0.16 | -0.21 | 0.17 | 0.04 |
| **H+Al** | -0.21 | -0.12 | 0.12 | -0.16 | 0.52 | 0.20 | -0.14 | -0.07 | 0.62 | 0.59 | 0.45 | 0.27 | 0.57 | 0.59 | -0.01 | 0.08 | 0.12 | 0.19 |
| **BD** | 0.11 | 0.18 | -0.18 | -0.05 | -0.46 | -0.21 | 0.31 | -0.03 | -0.76 | -0.77 | -0.58 | -0.55 | -0.26 | -0.36 | 0.26 | 0.08 | -0.37 | -0.27 |
| **SDC** | 0.80 | 0.86 | -0.86 | -0.24 | 0.03 | -0.23 | 0.67 | -0.50 | 0.03 | -0.09 | 0.15 | 0.03 | -0.12 | 0.10 | 0.14 | -0.03 | -0.50 | -0.36 |
| **RP** | 0.49 | 0.66 | -0.66 | -0.48 | 0.26 | 0.17 | 0.60 | -0.28 | 0.28 | 0.22 | 0.39 | 0.22 | 0.46 | 0.50 | 0.37 | 0.07 | -0.33 | -0.10 |
| **MaP** | -0.80 | -0.87 | 0.87 | 0.34 | 0.20 | 0.07 | -0.78 | 0.35 | 0.05 | 0.11 | -0.11 | -0.04 | -0.01 | -0.18 | -0.24 | 0.01 | 0.33 | 0.19 |
| **MiP** | 0.84 | 0.83 | -0.83 | -0.24 | 0.27 | 0.03 | 0.57 | -0.47 | 0.73 | 0.66 | 0.72 | 0.60 | 0.22 | 0.54 | -0.01 | -0.14 | -0.05 | 0.01 |
| **TP** | -0.06 | -0.13 | 0.13 | 0.12 | 0.46 | 0.11 | -0.29 | -0.08 | 0.76 | 0.75 | 0.57 | 0.53 | 0.21 | 0.34 | -0.29 | -0.14 | 0.31 | 0.23 |
| **WFPS** | 1.00 | 0.94 | -0.94 | -0.19 | 0.01 | -0.16 | 0.70 | -0.59 | 0.32 | 0.21 | 0.40 | 0.31 | -0.07 | 0.28 | 0.01 | -0.15 | -0.26 | -0.15 |
| **SWSC** |  | 1.00 | -1.00 | -0.27 | 0.04 | -0.14 | 0.77 | -0.59 | 0.32 | 0.21 | 0.42 | 0.30 | 0.07 | 0.38 | 0.12 | -0.11 | -0.32 | -0.16 |
| **SAC** |  |  | 1.00 | 0.27 | -0.04 | 0.14 | -0.77 | 0.59 | -0.32 | -0.21 | -0.42 | -0.30 | -0.07 | -0.38 | -0.12 | 0.11 | 0.32 | 0.16 |
| **K_fs_** |  |  |  | 1.00 | -0.17 | -0.63 | -0.38 | -0.20 | -0.25 | -0.25 | -0.33 | -0.27 | -0.36 | -0.47 | -0.20 | -0.21 | 0.00 | 0.01 |
| **AGS** |  |  |  |  | 1.00 | 0.38 | -0.26 | 0.02 | 0.56 | 0.42 | 0.52 | 0.54 | 0.01 | 0.33 | -0.02 | 0.24 | -0.05 | -0.09 |
| **MWD** |  |  |  |  |  | 1.00 | -0.09 | 0.67 | 0.37 | 0.40 | 0.43 | 0.54 | 0.41 | 0.52 | 0.16 | 0.33 | 0.40 | 0.27 |
| **VESS** |  |  |  |  |  |  | 1.00 | -0.39 | 0.12 | 0.11 | 0.29 | 0.11 | 0.29 | 0.32 | 0.18 | -0.09 | -0.32 | -0.18 |
| **SSI** |  |  |  |  |  |  |  | 1.00 | -0.01 | 0.10 | 0.04 | 0.19 | 0.17 | -0.06 | 0.07 | 0.37 | 0.47 | 0.24 |
| **SOC** |  |  |  |  |  |  |  |  | 1.00 | 0.97 | 0.91 | 0.80 | 0.46 | 0.67 | -0.11 | 0.00 | 0.21 | 0.16 |
| **TN** |  |  |  |  |  |  |  |  |  | 1.00 | 0.88 | 0.76 | 0.57 | 0.66 | -0.08 | -0.06 | 0.31 | 0.26 |
| **MBC** |  |  |  |  |  |  |  |  |  |  | 1.00 | 0.91 | 0.45 | 0.70 | -0.07 | 0.05 | 0.13 | 0.09 |
| **MBN** |  |  |  |  |  |  |  |  |  |  |  | 1.00 | 0.26 | 0.62 | -0.04 | 0.07 | 0.16 | 0.10 |
| **BG** |  |  |  |  |  |  |  |  |  |  |  |  | 1.00 | 0.70 | 0.28 | 0.03 | 0.27 | 0.38 |
| **AcP** |  |  |  |  |  |  |  |  |  |  |  |  |  | 1.00 | 0.21 | 0.03 | 0.22 | 0.34 |
| **Eworm** |  |  |  |  |  |  |  |  |  |  |  |  |  |  | 1.00 | 0.31 | 0.03 | 0.25 |
| **Mdens** |  |  |  |  |  |  |  |  |  |  |  |  |  |  |  | 1.00 | 0.07 | -0.19 |
| **Mrich** |  |  |  |  |  |  |  |  |  |  |  |  |  |  |  |  | 1.00 | 0.86 |

Pearson’s correlation coefficients significant at *p*<0.01 and *p*<0.05 are highlighted in dark gray and light gray cells, respectively; non-significant values are in white cells.

^§^P: phosphorus, S: sulfur, K: potassium, Ca: calcium, Mg: magnesium, B: boron, Cu: cooper, Fe: iron, Mn: manganese, Zn: zinc, CEC_pH7_: potential cation exchange capacity, H+Al: potential acidity, pH: potential of hydrogen in solution of CaCl_2_ 0.01 mol L^-1^ (1:2.5), BS: base saturation, BD: bulk density, SDC: soil degree of compactness, SRP: soil resistance to penetration, MaP: macroporosity, MiP: microporosity, TP: total porosity, WFPS: water-filled pore space, SWSC: soil water storage capacity, SAC: soil aeration capacity, K_fs_: field-saturated hydraulic conductivity; AGS: macroaggregation (>250µm) stability, MWD: mean weight diameter, VESS: visual evaluation of soil structure, SSI: structural stability index, SOC: soil organic carbon, TN: total nitrogen, MBC: microbial biomass carbon, MBN: microbial biomass nitrogen, BG:β Glucosidase activity, AcP: acid phosphatase activity, Eworm: number of earthworm, MDens: macrofauna density, MRich: macrofauna richness and MDiver: macrofauna diversity.
