# Supplementary material for: Soil Quality Indexing Strategies for Evaluating Sugarcane Expansion in Brazil
Source: PLoS One. 2016 Mar 3;11(3):e0150860. doi: 10.1371/journal.pone.0150860 (PMC4777567; doi:10.1371/journal.pone.0150860)
Supplement: S2 Table — (DOCX) [file pone.0150860.s002.docx]

**Electronic supporting information**

**S2 Table**. Model of soil functions framework and indicators^§^ used to develop the SQI-2.

| Soil Functions | Weight | Soil Indicators | | | | | | Transformed Indicator value^†^ | Indicator score | Soil function score | Weighted soil function score | **SQI** |
| --- | --- | --- | --- | --- | --- | --- | --- | --- | --- | --- | --- | --- |
|  |  | Level 1 | Weight | Level 2 | Weight | Level 3 | Weight |  |  | ∑(V*IV*III*II) |  |  |
|  | **I** |  | **II** |  | **III** |  | **IV** | **V** | (V*IV*III*II) | **VI** | (VI*I) | ∑(VI*I) |
| **F(i) - Storage, availability and cycling of nutrients** | 0.20 | Nutrient availability | 0.40 | Macronutrients | 0.80 | TN | 0.20 | 0.44 | 0.02816 | 0.900 | 0.180 | **0.848** |
|  |  |  |  |  |  | P | 0.20 | 0.86 | 0.05504 |  |  |  |
|  |  |  |  |  |  | K | 0.15 | 1.00 | 0.04800 |  |  |  |
|  |  |  |  |  |  | Ca | 0.15 | 1.00 | 0.04800 |  |  |  |
|  |  |  |  |  |  | Mg | 0.15 | 1.00 | 0.04800 |  |  |  |
|  |  |  |  |  |  | S | 0.15 | 0.93 | 0.04464 |  |  |  |
|  |  |  |  | Micronutrients | 0.20 | B | 0.20 | 0.92 | 0.01472 |  |  |  |
|  |  |  |  |  |  | Cu | 0.20 | 1.00 | 0.01600 |  |  |  |
|  |  |  |  |  |  | Mn | 0.20 | 1.00 | 0.01600 |  |  |  |
|  |  |  |  |  |  | Fe | 0.20 | 1.00 | 0.01600 |  |  |  |
|  |  |  |  |  |  | Zn | 0.20 | 1.00 | 0.01600 |  |  |  |
|  |  | Acidity/Al toxicity | 0.40 | pH | 0.25 |  |  | 0.96 | 0.09600 |  |  |  |
|  |  |  |  | H+Al | 0.25 |  |  | 1.00 | 0.10000 |  |  |  |
|  |  |  |  | BS | 0.50 |  |  | 1.00 | 0.20000 |  |  |  |
|  |  | Nutrient storage and cycling | 0.15 | CEC_pH7_ | 0.40 |  |  | 0.78 | 0.04680 |  |  |  |
|  |  |  |  | SOM | 0.60 | SOC | 0.50 | 0.41 | 0.01845 |  |  |  |
|  |  |  |  |  |  | MBC | 0.25 | 1.00 | 0.02250 |  |  |  |
|  |  |  |  |  |  | MBN | 0.25 | 0.95 | 0.02138 |  |  |  |
|  |  | Nutrient cycling | 0.05 | Enzyme activity | 1.00 | AcP | 0.50 | 0.90 | 0.02250 |  |  |  |
|  |  |  |  |  |  | BG | 0.50 | 0.86 | 0.02150 |  |  |  |
| **F(ii) - Infiltration, storage and availability of water, and soil aeration** | 0.20 | Water infiltration | 0.25 | Kfs | 0.70 |  |  | 1.00 | 0.17500 | 0.676 | 0.135 |  |
|  |  |  |  | Correlated indicators | 0.30 | SOC | 0.20 | 0.41 | 0.00615 |  |  |  |
|  |  |  |  |  |  | BD | 0.50 | 1.00 | 0.03750 |  |  |  |
|  |  |  |  |  |  | Eworm | 0.30 | 0.19 | 0.00428 |  |  |  |
|  |  | Water storage and availability | 0.25 | SWSC | 0.50 |  |  | 0.42 | 0.05250 |  |  |  |
|  |  |  |  | WFPS | 0.30 |  |  | 0.65 | 0.04875 |  |  |  |
|  |  |  |  | MiP | 0.10 |  |  | 0.91 | 0.02275 |  |  |  |
|  |  |  |  | Correlated indicator | 0.10 | TP | 1.00 | 1.00 | 0.02500 |  |  |  |
|  |  | Soil aeration | 0.50 | SAC | 0.45 |  |  | 0.13 | 0.02925 |  |  |  |
|  |  |  |  | MaP | 0.45 |  |  | 1.00 | 0.22500 |  |  |  |
|  |  |  |  | Correlated indicator | 0.10 | TP | 1.00 | 1.00 | 0.05000 |  |  |  |
| **F(iii) - Sustain biological activity** | 0.20 | SOC | 0.10 |  |  |  |  | 0.41 | 0.04100 | 0.681 | 0.136 |  |
|  |  | Microbial biomass | 0.30 | MBC | 0.50 |  |  | 1.00 | 0.15000 |  |  |  |
|  |  |  |  | MBN | 0.50 |  |  | 0.95 | 0.14250 |  |  |  |
|  |  | Edaphic macrofauna | 0.40 | Eworm | 0.10 |  |  | 0.19 | 0.00760 |  |  |  |
|  |  |  |  | Mdens | 0.20 |  |  | 1.00 | 0.08000 |  |  |  |
|  |  |  |  | Mrich | 0.30 |  |  | 0.79 | 0.09480 |  |  |  |
|  |  |  |  | Mdiver | 0.40 |  |  | 0.69 | 0.11040 |  |  |  |
|  |  | Correlated Indicators | 0.20 | SWSC | 0.50 |  |  | 0.42 | 0.04200 |  |  |  |
|  |  |  |  | SAC | 0.50 |  |  | 0.13 | 0.01300 |  |  |  |
| **F(iv)- Sustain the plant growth** | 0.20 | VESS | 0.20 |  |  |  |  | 0.98 | 0.19600 | 0.984 | 0.197 |  |
|  |  | SRP | 0.20 |  |  |  |  | 1.00 | 0.20000 |  |  |  |
|  |  | Soil compaction | 0.50 | BD | 0.50 |  |  | 1.00 | 0.25000 |  |  |  |
|  |  |  | 0.50 | SDC | 0.50 |  |  | 1.00 | 0.25000 |  |  |  |
|  |  | Correlated Indicators | 0.10 | SOC | 0.20 |  |  | 0.41 | 0.00820 |  |  |  |
|  |  |  | 0.10 | AGS | 0.40 |  |  | 1.00 | 0.04000 |  |  |  |
|  |  |  | 0.10 | TP | 0.40 |  |  | 1.00 | 0.04000 |  |  |  |
| **F(v)-Ability to resist degradation** | 0.20 | Structural stability | 0.60 | SSI | 0.50 |  |  | 1.00 | 0.30000 | 1.000 | 0.200 |  |
|  |  |  |  | AGS | 0.25 |  |  | 1.00 | 0.15000 |  |  |  |
|  |  |  |  | MWD | 0.25 |  |  | 1.00 | 0.15000 |  |  |  |
|  |  | Water infiltration | 0.40 | Kfs | 1.00 |  |  | 1.00 | 0.40000 |  |  |  |

^§^Abbreviations are same as S1 Table. ^†^Indicator value obtained by non-linear transformation of measured values, as described in the second step of soil quality index calculation.
